# PerSort Facilitates Characterization and Elimination of Persister Subpopulation in Mycobacteria

**DOI:** 10.1128/mSystems.01127-20

**Published:** 2020-12-01

**Authors:** Vivek Srinivas, Mario L. Arrieta-Ortiz, Amardeep Kaur, Eliza J. R. Peterson, Nitin S. Baliga

**Affiliations:** a Institute for Systems Biology, Seattle, Washington, USA; b Department of Biology, University of Washington, Seattle, Washington, USA; c Department of Microbiology, University of Washington, Seattle, Washington, USA; d Molecular Engineering Program, University of Washington, Seattle, Washington, USA; e Lawrence Berkeley National Lab, Berkeley, California, USA; UCSF

**Keywords:** *Mycobacterium*, phenotypic heterogeneity, persisters, antibiotic tolerance, nutrient starvation

## Abstract

Mycobacterium tuberculosis (MTB) generates phenotypic diversity to persist and survive the harsh conditions encountered during infection. MTB avoids immune effectors and antibacterial killing by entering into distinct physiological states. The surviving cells, persisters, are a major barrier to the timely and relapse-free treatment of tuberculosis (TB). We present for the first time, PerSort, a method to isolate and characterize persisters in the absence of antibiotic or other pressure. We demonstrate the value of PerSort to isolate translationally dormant cells that preexisted in small numbers within *Mycobacterium* species cultures growing under optimal conditions but that dramatically increased in proportion under stress conditions. The translationally dormant subpopulation exhibited multidrug tolerance and regrowth properties consistent with those of persister cells. Furthermore, PerSort enabled single-cell transcriptional profiling that provided evidence that the translationally dormant persisters were generated through a variety of mechanisms, including *vapC30*, *mazF*, and *relA*/*spoT* overexpression. Finally, we demonstrate that notwithstanding the varied mechanisms by which the persister cells were generated, they converge on a similar low-oxygen metabolic state that was reversed through activation of respiration to rapidly eliminate persisters fostered under host-relevant stress conditions. We conclude that PerSort provides a new tool to study MTB persisters, enabling targeted strategies to improve and shorten the treatment of TB.

**IMPORTANCE** Mycobacterium tuberculosis (MTB) persists and survives antibiotic treatments by generating phenotypically heterogeneous drug-tolerant subpopulations. The surviving cells, persisters, are a major barrier to the relapse-free treatment of tuberculosis (TB), which is already killing >1.8 million people every year and becoming deadlier with the emergence of multidrug-resistant strains. This study describes PerSort, a cell sorting method to isolate and characterize, without antibiotic treatment, translationally dormant persisters that preexist in small numbers within *Mycobacterium* cultures. Characterization of this subpopulation has discovered multiple mechanisms by which mycobacterial persisters emerge and unveiled the physiological basis for their dormant and multidrug-tolerant physiological state. This analysis has discovered that activating oxygen respiratory physiology using l-cysteine eliminates preexisting persister subpopulations, potentiating rapid antibiotic killing of mycobacteria under host-relevant stress. PerSort serves as a new tool to study MTB persisters for enabling targeted strategies to improve and shorten the treatment of TB.

## Abstract (precis)

Mycobacterium tuberculosis (MTB) persists and survives antibiotic treatments by generating phenotypically heterogeneous drug-tolerant subpopulations. The surviving cells, persisters, are a major barrier to the relapse-free treatment of tuberculosis (TB), which is already killing >1.8 million people every year and becoming deadlier with the emergence of multidrug-resistant strains.

## INTRODUCTION

Studies have revealed cell-to-cell variability in organisms from all domains of life—unicellular to multicellular. Through cell-to-cell variability, cell populations are prepared for sudden environmental changes by harboring subpopulations that are phenotypically preadapted. This evolutionary strategy, known as bet-hedging (1), confers fitness advantage to pathogens, such as Mycobacterium tuberculosis (MTB), which routinely experience varying environments within the host. In addition to spontaneous bet-hedging, MTB responds to host-derived stresses with physiological changes (e.g., shifts in metabolism and respiration, induction of toxin-antitoxin systems, cell wall modifications) that allow it to survive and persist. These physiological changes (either stochastically or environmentally induced) result in antibiotic tolerance, in which MTB is genetically susceptible to antibiotics but exists in a physiological state rendering it refractory to drug killing. These persistent states are a major reason why long courses of antibiotic therapy are required to treat human tuberculosis (TB) (2); standard chemotherapy of TB requires 6 months of treatment, and 5% are not cured even then (3, 4). There is an urgent need for new strategies that shorten the duration of treatment and target drug-tolerant MTB. Addressing this gap requires a better understanding of how MTB generates phenotypic heterogeneity to withstand pressures from the host environment and evade antibiotic therapy.

Recent work has studied phenotypic heterogeneity in MTB from single-cell analyses using fluorescent reporter strains. Cell-to-cell variability of MTB was captured *in vitro* and during murine infections using a reporter of 16S rRNA gene expression (5). The microscopy-based platform was able to track heterogeneity in growth rate under standard growth conditions and found that heterogeneity was amplified by stress conditions and murine infection. However, the nongrowing subpopulation was not isolated and characterized further, most likely due to low fluorescence levels of the reporter. In another study, Jain et al. developed a dual-reporter mycobacteriophage (φ^2^DRM) system and used fluorescence-activated cell sorting (FACS) to isolate drug-tolerant MTB cells from *in vitro* cultures and human sputa (6). However, the necessity to reinfect daughter cells with φ^2^DRM limited the ability to monitor isolated cells over generations and study their regrowth patterns. The study also used a reporter that specifically enriched for persisters of isoniazid treatment (i.e., fluorescent protein fused to the *dnaK* promoter), disregarding multidrug-tolerant subpopulations which are known to exist within the host environment (7).

Here, we sought to develop a fluorescent reporter system to isolate and characterize multidrug-tolerant subpopulations of mycobacteria from naive growth conditions (i.e., absence of antibiotic treatment). We wanted to avoid killing susceptible cells with antibiotics in order to study both persister cells and actively growing cells from the same culture and prevent drug pressure from confounding persister formation. Instead, on the basis of a considerable body of work linking bacterial stress pathways to the acquisition of drug tolerance via translation inhibition (8–13), we developed “PerSort” to enrich for translationally dormant mycobacteria (i.e., cells that are able to transcribe but not translate a fluorescent reporter) under naive conditions. We demonstrated the persister-like properties of the translationally dormant subpopulation and their increased abundance during stress conditions. Moreover, we performed single-cell transcriptional profiling of “PerSorted” cells and highlight various mechanisms that generate translationally dormant mycobacteria. Finally, using these mechanistic insights, we revealed a physiological response common to persister cells and demonstrated that activating respiration potentiated both isoniazid and rifampicin to rapidly clear mycobacteria. Importantly, this was achieved from a host-relevant condition that fosters multidrug-tolerant mycobacteria. This study confirms that PerSort enables a better understanding of mycobacterial phenotypic heterogeneity and can lead to new strategies for shortening TB treatment.

## RESULTS

### Multidrug-tolerant mycobacteria increase in nutrient-starved conditions and show lag dormancy.

**FIG 1:**
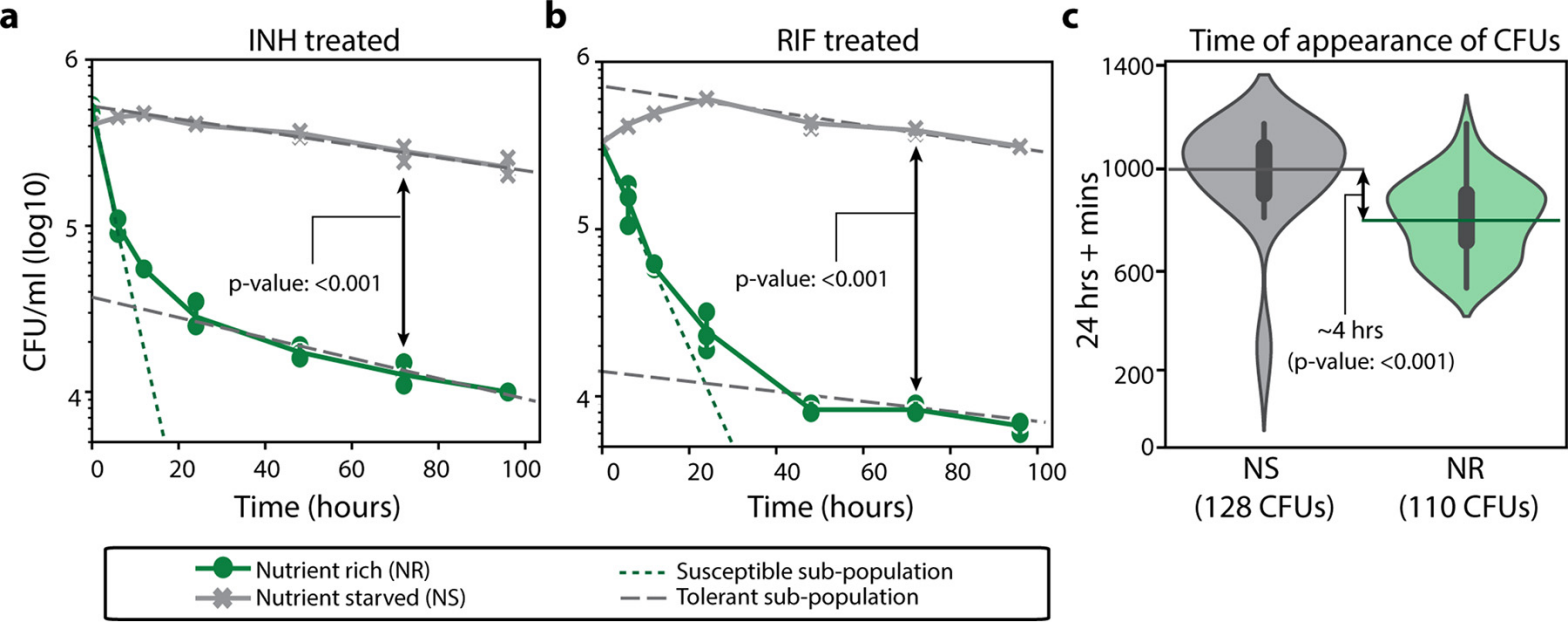
Time-kill curves and growth characteristics of M. smegmatis from nutrient-rich and nutrient-starved conditions. (a and b) Time-kill curves of MSM cultures grown in nutrient-rich (NR) or nutrient-starved (NS) conditions treated with 5× MIC isoniazid (INH) (a) or 5× MIC rifampicin (RIF) (b). The solid lines indicate experimentally observed time-kill curves. The dashed and dotted lines distinguish the slopes of the susceptible and tolerant subpopulations, respectively. Data points are from three experimental replicates; error bars were calculated by measuring standard deviation between replicates. The limit of quantification for reporting growth was 10^3^ CFU/ml. (c) ScanLag analysis showing time of appearance (TOA) of cultures grown in NR and NS conditions. Error bars within the violin plot are standard deviations with a confidence interval of 0.9. The dashed lines indicate the mean TOA from cultures grown in NR or NS conditions. Mean CFU/ml at 72 h and TOA between NS and NR conditions were compared with Student’s *t* test.

To establish a system that enriches for multidrug-tolerant mycobacteria, we grew Mycobacterium smegmatis (MSM) in nutrient-rich (7H9 medium, 0.2% glycerol, 0.05% Tween 80, and albumin-dextrose-catalase [ADC] complementation) and nutrient-starved (phosphate-buffered saline [PBS] and 0.05% Tween 80) conditions. Deprivation of nutrients results in a marked slowing of mycobacterial growth and concurrent phenotypic tolerance to various antibiotics, including isoniazid (INH) and rifampicin (RIF) (14, 15). Moreover, studies have established the relevance of the bacterial physiological state adopted under nutrient-starved conditions to TB infection (16). We incubated wild-type MSM mc^2^155 strain in nutrient-rich or nutrient-starved conditions for 12 h and then treated the strain with either 5× MIC of INH or RIF (20 and 17.5 μg/ml, respectively). The antibiotic-treated MSM cultures were diluted and plated over a 20-h interval, and colonies were enumerated to generate time-kill curves (Fig. 1a and b). According to the bimodal model of bacterial persistence (17), multiple subpopulations were identified, either drug susceptible or drug tolerant, as distinguished by various slopes of killing. We further estimated the abundance of drug-tolerant subpopulations in either nutrient-rich or nutrient-starved conditions via the intersection of the *y* axis with the extrapolated slope of the drug-tolerant subpopulation. Thus, MSM cultures grown in nutrient-rich conditions were ∼5% tolerant to INH and RIF. Whereas after nutrient starvation, almost the entirety of the population exhibited tolerance to INH and RIF.

Another advantage of the nutrient deprivation model for enriching and characterizing multidrug-tolerant mycobacteria is that nutrient-starved bacteria can easily grow upon being returned to nutrient-rich media; thus, this model allows easy quantification of drug susceptibility and growth patterns. We characterized the population-wide growth characteristics of the nutrient-rich and nutrient-starved cultures using ScanLag, a technique that combines cell plating with high-throughput imaging (18). Following 12 h of incubation in nutrient-rich or nutrient-starved conditions, MSM mc^2^155 cultures were plated onto nutrient-rich plates (7H10 medium, 0.2% glycerol, oleic acid-albumin-dextrose-catalase [OADC]) and observed for colony growth by imaging at 1 h intervals. The mean time of appearance (TOA) of individual colony-forming units (CFU) from the nutrient-rich conditions was determined to be 37 h, whereas the TOA from the nutrient-starved conditions was 41 h (Fig. 1c). This significant delay in resuming growth is due to a greater abundance of cells with lag dormancy, a phenotype well-established with drug tolerance (19, 20), which explains why nutrient-deprived mycobacteria are multidrug tolerant.

### PerSort isolates translationally dormant mycobacteria that are multidrug tolerant.

To further characterize multidrug-tolerant subpopulations, we developed a FACS-based protocol to isolate translationally dormant mycobacterial cells. In drug tolerance-inducing conditions, translation is repressed via various mechanisms that downregulate rRNA (e.g., RelA and CarD) or degrade rRNA (e.g., VapC and other toxins) (10–15). Therefore, we hypothesized that a fluorescent reporter that relays translation activity could efficiently sort and isolate drug-tolerant mycobacterium that form in a multitude of ways. The reporter plasmid (Trans-mEos2 [see Fig. S1 in the supplemental material]) was constructed by inserting the highly stable fluorescent gene, *mEos2*, under the transcriptional control of an anhydrotetracycline (ATc)-inducible promoter and a strong mycobacterial translation initiation sequence (Shine-Dalgarno sequence) and then inserted into the PstKi plasmid (16, 21, 22). The PstKi plasmid contains an integrase gene and thus eliminates copy number variability among cells by integrating into the mycobacterium genome. Moreover, the ATc-inducible promoter achieves detectable fluorescence from a genome-integrated reporter while also allowing for modulation of reporter expression.

10.1128/mSystems.01127-20.1 FIG S1 Plasmid map of Trans-mEos2. Tet promoter, tetracycline-inducible promoter; MYC SD, Shine-Dalgarno sequence of *Mycobacterium* spp.; ORI, origin of replication; KanR, kanamycin resistance cassette. Download FIG S1, JPG file, 0.1 MB. Copyright © 2020 Srinivas et al. 2020 Srinivas et al. This content is distributed under the terms of the Creative Commons Attribution 4.0 International license.

The Trans-mEos2 plasmid was transformed into wild-type MSM (creating the MSM-mEos2 strain) for differentiation of mEos2 fluorescence levels in mycobacterial cells using FACS. To minimize clumps, MSM-mEos2 cultures (grown in 7H9, 0.2% glycerol, 0.05% Tween 80, ADC complementation, and high shaking conditions) were passed through a 10-μm filter before running on the BD FACS Influx instrument. Parameters for single cells were defined by 3-μm rainbow fluorescent beads, and the efficiency of single-cell sorting was assessed using a mixed culture of ATc-induced MSM-mEos2 cells (resistant to kanamycin) and MSM cells expressing mCherry (resistant to hygromycin) (23). Cells from the mixed fluorescent reporter cultures were sorted and plated onto 7H10 plates with appropriate antibiotic selection. Sorting was found to be 93% efficient (Fig. S2).

10.1128/mSystems.01127-20.2 FIG S2 Efficiency in bacterial single-cell sorting. Bacterial cells (*n *= 2,000) from the equal-proportional mixture of MSM-mCherry strain (with mCherry fluorescence and hygromycin [HYG] resistance) and MSM-mEos2 strain (with mEos2 fluorescence and kanamycin [KAN] resistance) were sorted based on mCherry and mEos2 fluorescence. Cells (*n *= 2,000) from the mCherry sort and mEos2 sorts were plated onto both 7H10+HYG and 7H10+KAN plates to determine the percentage of MSM-mEos2 cells in mCherry sort and MSM-mCherry cells in mEos2 sort (i.e., inappropriately sorted). Approximately 120 colony-forming units (CFUs) of MSM-mCherry were enumerated in the mEos2 sort (HYG plates, right panel) and 150 MSM-mEos2 cells in mCherry sort (KAN plates, right panel). This suggests that only ∼7% of cells were inappropriately sorted and that the sorting efficiency for single bacterial cells was ∼93%. Download FIG S2, JPG file, 0.1 MB. Copyright © 2020 Srinivas et al. 2020 Srinivas et al. This content is distributed under the terms of the Creative Commons Attribution 4.0 International license.

**FIG 2:**
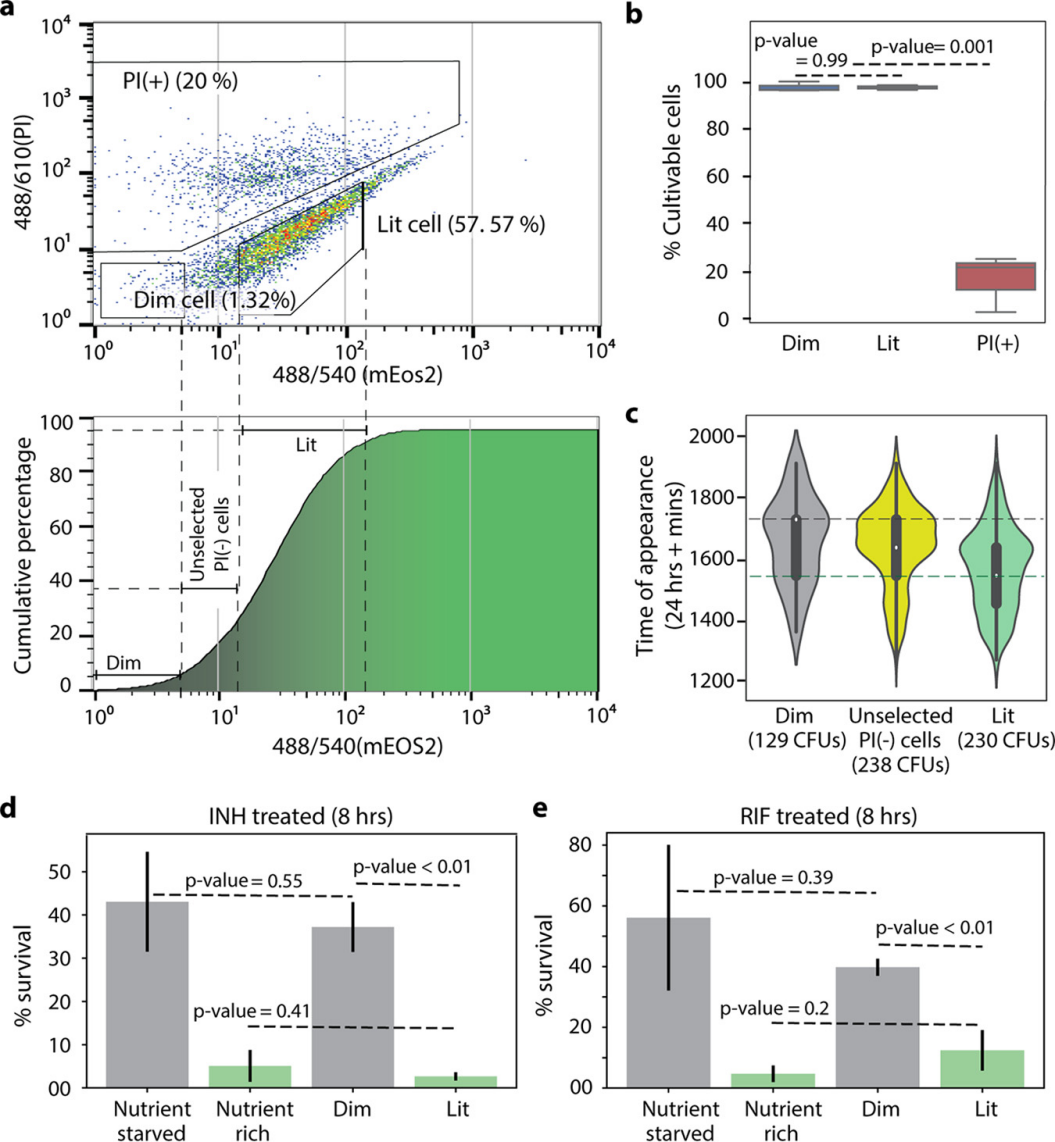
PerSort isolation and characterization of subpopulations from anhydrotetracycline (ATc)-induced MSM-mEos2 cultures. (a, top) Population structure of ATc-induced (500 ng/ml) MSM-mEos2 cells from single-cell gates, represented by mEos2 fluorescence on the *x* axis and propidium iodide (PI) (5 μl/ml) fluorescence on the *y* axis. Polygons indicate the gates for nonculturable cells [PI(+)], dim cells, and lit cells with the proportion of cells in that particular gate. (Bottom) Cumulative distribution of mEos2 florescence intensity in MSM-mEos2 cells, dashed lines indicate boundaries of sort gates for dim, lit, and unselected PI(-) cells. (b) Cultivability of PerSorted dim, lit, and PI(-) cells from ATc-induced MSM-mEos2 cultures. The percent cultivable cells was calculated from the number of CFU from 200 sorted cells. Experiments were performed in triplicates, and error bars represent the standard deviation between replicates. Significance of cultivability difference between dim, lit, and nonculturable subpopulations was calculated with Student’s *t* test. (c) ScanLag analysis of dim, unselected PI(-), and lit cells from PerSorted MSM-mEos2 cultures induced with ATc (500 ng/ml). The dashed lines in the violin plot indicate mean TOA of the sorted subpopulations. Error bars within the violin plot are standard deviations with a confidence interval of 0.9. (d and e) Percent survival of 5× MIC INH or 5× MIC RIF treatment of PerSorted dim and lit cells of MSM-mEos2 cultures induced with ATc (500 ng/ml), compared to percent survival of whole populations (i.e., unsorted) of MSM-mEos2 induced with ATc (500 ng/ml) and grown in nutrient-rich and starved conditions. Experiments were performed in triplicates, and error bars represent the standard deviation between replicates. Significance of survival between dim and lit subpopulations was calculated with Student’s *t* test.

Sort gates were defined for cultivable and noncultivable cells (i.e., dead cells and viable but nonculturable cells [VBNCs]). Viable but nonculturable cells are antibiotic tolerant, but they lack the ability to regain active growth in standard conditions and therefore do not contribute to bacterial survival (24). To exclude noncultivable cells, MSM-mEos2 cultures were stained with propidium iodide (PI), and PI signal was used for gate selection. Heat-killed cultures were used for evidence of nonculturable cells in the PI-positive [PI(+)] gate (Fig. S3). Gates for translationally active and dormant cells were defined based on mEos2 fluorescence signal in the PI-negative [PI(-)] cultivable population (Fig. 2a). Wild-type MSM and MSM-mEos2 cultures were grown in the presence of ATc (500 ng/ml) for 12 h and stained with 0.5% PI. Background fluorescence from wild-type MSM cells was used to define the gate for translationally dormant cells (“dim”). For clear distinction between subpopulations, gates were set at a higher mEoS2 fluorescence signal to define MSM-mEos2 translationally active cells (“lit”), leaving ∼25% of PI(-) cells unselected (Fig. 2a). Further, the PI signal gates were set such that thresholds for selection of cultivable cells, PI(-), decreased with the mEos2 signal, as VBNCs were expected at high proportions in low mEos2 signal range. Based on these FACS gates and following ATc induction of MSM-mEos2 cultures in standard 7H9 medium (i.e., same as nutrient-rich conditions) for 12 h (optical density at 600 nm [OD_600_] > 1), a majority of cells were translationally active “lit” cells, while a consistent subpopulation of translationally dormant “dim” cells was also present, reaching about 1% of the population (Fig. 2a). We analyzed mEos2 RNA levels in dim and lit cells by quantitative reverse transcription-PCR (qRT-PCR). Interestingly, we discovered that mEos2 RNA was expressed at a slightly higher level in dim cells than in lit cells (Fig. S4, mEos2 RNA log_2_ fold change = 1.03). This confirms that dim cells transcribe the reporter gene but fail to translate the RNA, thus resulting in low mEos2 fluorescence. Further, we sorted equal numbers of cells from the dim, lit, and PI(+) gates and plated onto 7H10 medium (Fig. 2b). All of the cells from the dim and lit subpopulations formed CFU, whereas only 25% formed CFU from the PI(+) subpopulation, confirming that cells from the PI(-) (i.e., dim and lit) gates were cultivable and devoid of VBNCs and dead cells. Finally, we measured dim cell proportions following ATc induction of MSM-mEos2 cultures in standard growth conditions until cultures were either in the exponential (∼6 h after ATc addition) or stationary (12 h after ATc addition) phase of growth. Dim cells were found in low numbers (∼0.4% of population) during exponential phase and increased during stationary phase to make up ∼1% of the population (Fig. S5). This indicates that dim cells preexist under low-stress conditions (i.e., exponential phase) and a density-dependent increase in dim cell formation, likely caused by nutrient depletion of the culture during stationary phase (25).

10.1128/mSystems.01127-20.3 FIG S3 (a) Gating strategies used in identification of translationally dormant (dim) cells. Gating between PI-stained live and dead MSM-mEos2 strains (left panel), and PI-stained nonfluorescing uninduced and ATc (500 ng/ml)-induced MSM-mEos2 strains (right panel). (b) mEos2 reporter expression in PerSorted MSM-mEos2 dim and translationally active (lit) subpopulation. Download FIG S3, JPG file, 0.1 MB. Copyright © 2020 Srinivas et al. 2020 Srinivas et al. This content is distributed under the terms of the Creative Commons Attribution 4.0 International license.

10.1128/mSystems.01127-20.4 FIG S4 Log_2_ normalized expression of mEos2 transcripts measured with qRT-PCR in PerSorted dim and lit cells (*n* = 300 cells). Error bars were calculated by measuring standard deviations of gene expression. *P* values were calculated with Student’s *t* test between the dim and lit cells. Download FIG S4, JPG file, 0.04 MB. Copyright © 2020 Srinivas et al. 2020 Srinivas et al. This content is distributed under the terms of the Creative Commons Attribution 4.0 International license.

10.1128/mSystems.01127-20.5 FIG S5 Translationally dormant (dim) cell percentages in exponential and stationary-phase cultures of MSM-mEos2 cultures induced with 500 ng/ml ATc at an OD_600_ of 0.05 and 0.6, respectively. Both samples were induced with ATc for 12 h at 37°C before they were PerSorted for dim cell percentages. Experiments were performed in triplicates, and error bars were calculated by measuring standard deviations in dim cell percentages from the three replicates. Download FIG S5, JPG file, 0.04 MB. Copyright © 2020 Srinivas et al. 2020 Srinivas et al. This content is distributed under the terms of the Creative Commons Attribution 4.0 International license.

Having developed the fluorescent reporter system and optimized the FACS procedure to sort translationally active and dormant cells from naive growth conditions (i.e., absence of antibiotic treatment), we investigated the growth characteristics and drug susceptibility of the dim and lit subpopulations. For growth characterization, cells were sorted from the PI(-) gate of ATc-induced MSM-mEos2 cultures and ScanLag analysis was performed as described previously (Fig. 2c). The dim cells showed a longer TOA compared to lit cells, with a difference between the subpopulations (∼200 min) similar to that observed between nutrient-rich and nutrient-starved cultures (Fig. 1c). As expected, cells from the unselected PI(-) gate had a mean TOA in between the dim and lit subpopulations, suggesting that it is almost an equal mixture of translationally active and inactive cells. To assess drug susceptibility, 100,000 dim and lit cells from ATc-induced MSM-mEos2 cultures were sorted into 7H9 medium containing either 5× MIC INH or 5× MIC RIF. The survival of dim and lit subpopulations was determined by comparing CFU after 12-h INH or 8-h RIF treatment to CFU before drug treatment (Fig. 2d and e). The drug treatment times were selected from the slowed phase of killing from the biphasic kill curves (Fig. 1a and b). A significantly larger percentage of dim cells survived both INH (*P* value = 0.001) and RIF treatment (*P* value = 0.006), indicating that the dim cells are a multidrug-tolerant subpopulation. Further, the survival of dim cells was compared to the entire population of ATc-induced MSM-mEos2 cultures (i.e., unsorted) grown in either nutrient-rich or nutrient-starved conditions. The percentage of dim cells surviving INH and RIF treatment matched closely with the nutrient-starved cultures. These results reveal the striking similarities, both in terms of growth characteristics (i.e., lag dormancy) and antibiotic susceptibility (i.e., multidrug tolerance), between dim cells and nutrient-starved cultures. As such, we hypothesized the dim cells are a phenotypically heterogeneous subpopulation, with translational dormancy and multidrug tolerance, that increase in proportion under nutrient deprivation because their physiological state is best suited to withstand such environmental stress.

### Translationally dormant mycobacteria form at different probabilities in nutrient-rich versus nutrient-starved conditions.

**FIG 3:**
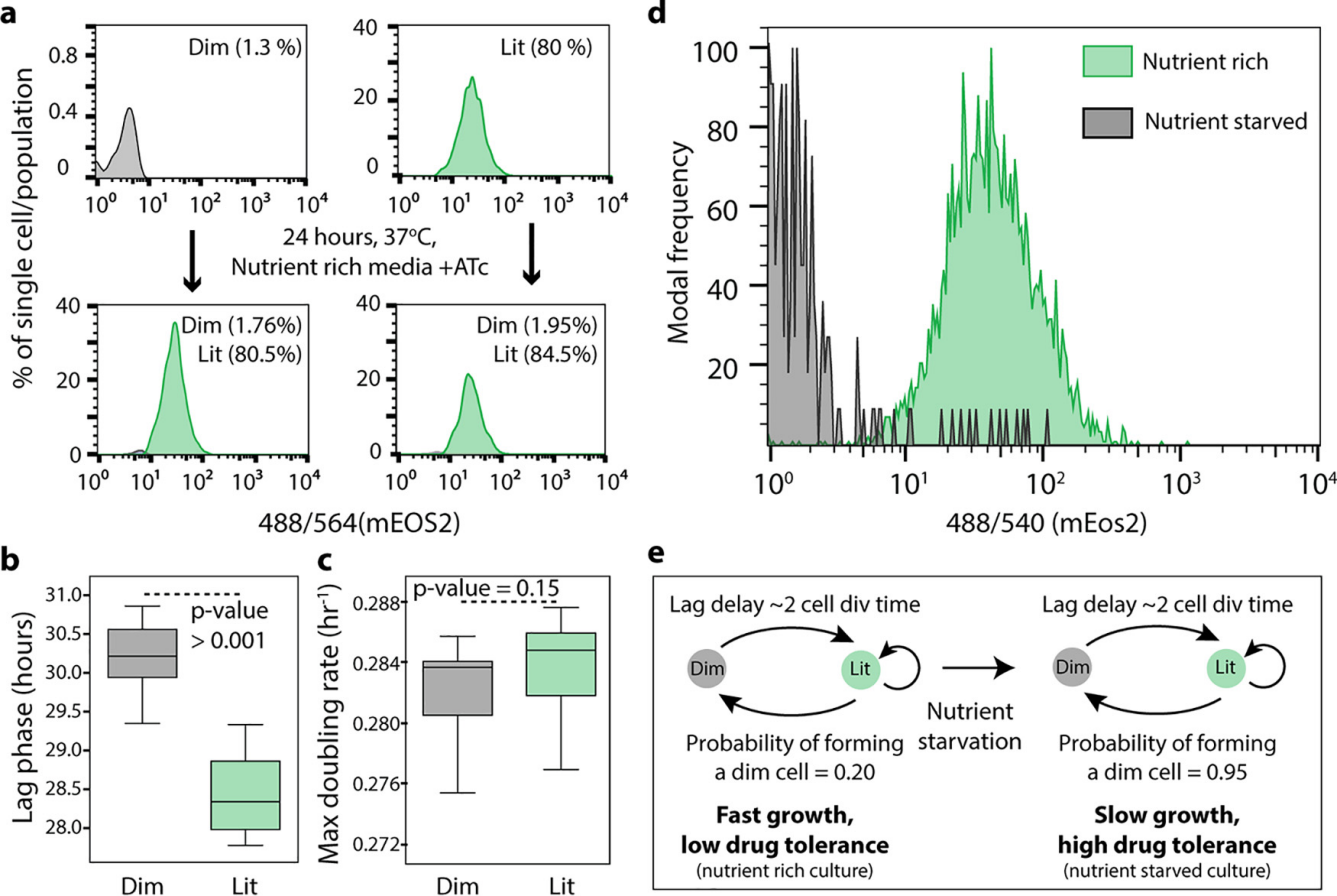
Regrowth dynamics of PerSorted dim and lit cells and population structure under nutrient-rich versus nutrient-starved conditions. (a) Dim and lit cell proportions in cultures regrown from PerSorted dim and lit populations obtained from ATc-induced MSM-mEos2 cultures. The proportions of cells in each subpopulation were measured from the gating methods described in the legend to Fig. 2a. (b and c) Lag phase and maximum growth rate were calculated from OD_600_ absorbance measurements of dim and lit cells PerSorted into 7H9 medium. Error bars were calculated by measuring standard deviations in the lag phase and maximum growth rates between dim and lit cells (*n *= 100,000). Significance of differences between dim and lit subpopulations was calculated with Student’s *t* test. (d) Population structure of ATc-induced MSM-mEos2 cultures grown in nutrient-rich (green) and nutrient-starved (gray) conditions. (e) A model describing the formation of dim cells under nutrient-rich and nutrient-starved conditions in mycobacterium. The probability of dim cell formation was calculated from their relative proportion in the indicated culture conditions.

We used the demonstrated capability of PerSort to isolate persister-like mycobacterial cells to investigate the population structure and regrowth characteristics of subpopulations from both nutrient-rich (also devoid of antibiotic treatment) and nutrient-starved conditions. Following ATc induction of the MSM-mEos2 culture for 12 h, 100,000 dim and lit cells were PerSorted into standard 7H9 medium. Sorted samples were grown to an OD_600_ of 0.6, induced with ATc again for 12 h, and reanalyzed by FACS. Cultures generated from both dim and lit subpopulations had similar structure as the parent population vis-à-vis proportions of dim and lit cells (Fig. 3a). These results demonstrate that translational dormancy of dim cells is not heritable and that dim and lit cells interconvert under standard *in vitro* growth conditions. We further assessed regrowth by measuring OD_600_ over time after PerSorting 100,000 dim and lit cells into standard 7H9 medium. As expected, dim cell cultures grew after a longer lag phase than lit cells (Fig. 3b), but the subpopulations reached similar maximum growth rate and carrying capacity (Fig. 3c). This demonstrates that dim cells can resume normal growth (i.e., same as lit cells) in low-stress conditions, which is a key characteristic of persister cells (26). Further, ATc-induced MSM-mEos2 cultures were grown in either nutrient-rich or nutrient-starved conditions and then PerSorted to determine the population structure. Both dim and lit cells were present in the nutrient-starved cultures, but there was a significant increase in the proportion of dim cells (Fig. 3d). This confirmed our hypothesis, establishing that dim cells (i.e., translationally dormant mycobacteria) dramatically increase in proportion upon nutrient starvation, further suggesting that the likelihood of generating a dim cell in each cell division is distinct under low-stress versus high-stress conditions (Fig. 3e). Whereas dim cells (and likely other phenotypically heterogeneous subpopulations) form at low probability under nutrient-rich conditions, nutrient deprivation favors the formation of dim cells (a translationally dormant and multidrug-tolerant subpopulation) and enables mycobacteria to withstand stress, including drug treatment.

### Translationally dormant mycobacteria are composed of three discernible subtypes of *vapC30*, *mazF* toxins, and/or *relA*/*spoT* overexpressing cells.

To further characterize the formation of phenotypically heterogeneous mycobacteria, we profiled within individual cells of both dim and lit subpopulations following PerSort, the transcript levels of 45 genes that were previously implicated in persister formation and drug tolerance in *Mycobacterium* spp. and Escherichia coli (see Table S1 in the supplemental material). Single-cell gene expression profiling was performed with the Fluidigm Biomark 48 × 48 system per the manufacturer’s instructions and assayed relative to single-cell genomic DNA signal. The DNA signal from single dim and lit cells was found to have low variation in expression (Fig. S6a), indicating that the number of transcripts per genome copy accurately reports differential gene expression. Transcript abundances were normalized to a spike-in RNA control to account for experimental noise (Fig. S6b).

10.1128/mSystems.01127-20.6 FIG S6 Quality analysis of Fluidigm Biomark persister gene assay as shown by cycle of quantification (CT) histograms. (a) Genomic DNA expression in PerSorted dim and lit cells. The genomic region considered for measurement was from the PBR372 region of the pSTKi-mEos2 plasmid that is inserted into the genomic DNA of the MSM-mEos2 strain. (b) RNA spike in control from PerSorted dim and lit cells indicating uniformity in cell lysis and PCR efficiency, respectively. Download FIG S6, JPG file, 0.1 MB. Copyright © 2020 Srinivas et al. 2020 Srinivas et al. This content is distributed under the terms of the Creative Commons Attribution 4.0 International license.

10.1128/mSystems.01127-20.9 TABLE S1 Genes used in single-cell persister gene expression assays. Weights were assigned for their ability to differentiate between dim and lit cells and the respective primers used in single-cell persister gene expression assays. Download Table S1, DOCX file, 0.02 MB. Copyright © 2020 Srinivas et al. 2020 Srinivas et al. This content is distributed under the terms of the Creative Commons Attribution 4.0 International license.

**FIG 4:**
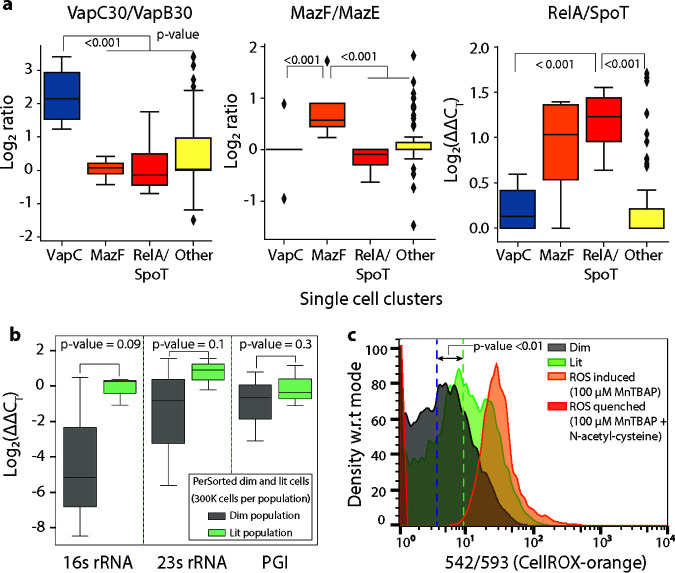
Single-cell gene expression and ROS levels from PerSorted single dim and lit cells. (a) Single-cell expression of key persister genes from clusters of dim cells identified from hierarchical clustering. The clusters were defined by their increased expression of specific persister genes. Other cells are single lit cells that do not form gene-specific clusters. The log_2_ ΔΔ*C_T_* (or ratio) is shown on the *y* axis. *P* values were calculated with Student’s *t* test between clusters of cells. (b) qRT-PCR measurement of 16S rRNA, 23S rRNA, and phosphoglucoisomerase (PGI) (MSMEG_5541) levels in PerSorted dim and lit cells (*n *= 300 cells). Error bars were calculated by measuring standard deviations of gene expression. *P* values were calculated with Student’s *t* test between the dim and lit cells. (c) Reactive oxygen species (ROS) levels, measured with CellRox, of ATc-induced MSM-mEos2 cultures grown in nutrient-rich conditions and PerSorted into dim and lit subpopulations. ROS was quenched with *N*-acetyl cysteine and induced with MnTBAP and used as reference controls. Significance of ROS levels between dim and lit subpopulations was calculated by Kolmogorov-Smirnov (K-S) test (K-S statistics of CellRox orange intensity between dim and lit population; K-S max difference, 56.1%, K-S max at intensity, 14.8551, K-S probability, >99.9%). w.r.t, with respect to.

Kernel principal-component analysis (kPCA) with radial basis function identified distinct clusters consisting of dim and lit cells as well as some overlap between the subpopulations (Fig. S7a). The overlap could indicate some phenotypic uncertainty, based on the expression of selected persister genes. We used a tree-based feature selection (see Materials and Methods) to rank the persister genes for their ability to differentiate the dim and lit subpopulations (27). Using the top features (Table S1), we performed unsupervised hierarchical clustering and dynamic tree cutting to identify four distinct clusters within the dim cells (Fig. S7b) (28, 29). Specifically, clustering revealed subtypes of translationally dormant mycobacteria with high *relA*/*spoT* expression, and high toxin/antitoxin ratios for *vapC30*/*vapB30* or *mazF*/*mazE* (and another subtype with no distinct signature of persister gene expression). Importantly, similar clustering analysis did not detect any statistically significant subtypes within the lit cells (Fig. S7b). Increased expression of *relA*/*spoT*, *vapC30*/*vapB30*, or *mazF*/*mazE* ratio was observed only within clusters of dim cells, not all single cells analyzed (Fig. 4a). This suggested that differential expression of these key persister genes in dim and lit cells would be undetectable by bulk expression profiling. As such, we isolated transcripts from PerSorted dim and lit cells (*n* = 300,000) and enriched them using the Path-seq methodology for bulk gene expression analysis (30). As expected, little or no variation was observed in the expression of the persister-specific genes (*relA*/*spoT*, *vapC30*/*vapB30*, or *mazF*/*mazE*) when analyzed in bulk (Fig. S8). This highlights the necessity of single-cell measurements to identify mechanisms by which heterogeneous subpopulations are formed.

10.1128/mSystems.01127-20.7 FIG S7 (a) kPCA plot that used radial basis function for dimensionality reduction of persister gene expression (ΔΔCT; 45 genes) in PerSorted single dim and lit cells isolated from MSM-mEos2 cultures induced with ATc (500 ng/ml). Solid lines in the scatter plot indicate the density estimates of the population (outliers were omitted in the plot). (b) Hierarchical clustering of top ranked features in PerSorted dim and lit single cells. Blocks in the cluster represent the statistically significant clusters (*P* value = 0.01). Download FIG S7, JPG file, 0.2 MB. Copyright © 2020 Srinivas et al. 2020 Srinivas et al. This content is distributed under the terms of the Creative Commons Attribution 4.0 International license.

10.1128/mSystems.01127-20.8 FIG S8 Expression of key persister specific genes from PerSorted bulk dim and lit cells (*n* = 300,000, replicates = 4). *P* values were calculated with Student’s *t* test between dim and lit cells. NS, not significant. Download FIG S8, JPG file, 0.1 MB. Copyright © 2020 Srinivas et al. 2020 Srinivas et al. This content is distributed under the terms of the Creative Commons Attribution 4.0 International license.

We sought further evidence that one or some combination of the three gene features identified from single-cell expression profiling (i.e., RelA/SpoT, VapC30, or MazF) were active in the dim cell subpopulation. The VapC30 and MazF toxins belong to a family of type II toxin-antitoxin (TA) systems, implicated in mycobacterial dormancy and persistence (25, 31, 32). Activation of the type II TA system results in toxin-mediated cleavage of tRNAs (1) or rRNAs (12, 33) causing translational dormancy (34). We analyzed 16S and 23S rRNA levels in dim and lit cells by qRT-PCR. We discovered that indeed dim cells have lower rRNA content relative to lit cells (Fig. 4b, 16S rRNA log_2_ fold change = −4.17, 23S rRNA log_2_ normalized fold change = −2.34). There was no discernible decrease in the transcript level of a highly expressed metabolic gene (Fig. 4b, phosphoglucoisomerase [PGI] log_2_ fold change = −0.87). These results support the notion that some of the translationally dormant mycobacteria are formed via toxin-mediated cleavage of rRNA.

### Translationally dormant mycobacteria are in a low-O_2_ respiratory state.

The single-cell expression data revealed that translationally dormant mycobacteria may be formed by at least three distinct mechanisms. The multiplicity of mechanisms confers robustness to the pathogen, but it also thwarts therapeutic strategies to block formation of the multidrug-tolerant mycobacteria. Nonetheless, we found evidence from the literature that the mechanisms for persister formation converge on a common physiology and predicted the dim cells are in a reduced state of respiration. Direct measurements of respiration rate (e.g., oxygen electrode or Seahorse analysis) are problematic with low bacterial cell numbers. Therefore, to test whether dim cells have lower O_2_ metabolism, we measured reactive oxygen species (ROS) levels in single cells from the PerSorted dim and lit subpopulations using CellRox orange. It is established that increases in ROS levels are linked with increased flux to the tricarboxylic acid (TCA) cycle and oxidative metabolism (35, 36). Cultures of ATc-induced MSM-mEos2 were grown in standard 7H9 medium, stained with CellRox orange (37), and PerSorted to compare ROS levels between dim and lit cells (Fig. 4c). As reference controls, MSM-mEos2 cultures were treated with manganese(III) tetrakis(4-benzoic acid) porphyrin chloride (Mn-TBAP) (ROS inducer) or *N*-acetyl-cysteine (ROS quencher) with Mn-TBAP. Cultures were incubated in CellRox orange stain for 1.5 h to ensure uniform dye permeation across the subpopulations. The ROS levels were significantly lower in dim cells (Fig. 4c), confirming that the translationally dormant mycobacteria have reduced oxidative metabolism. Furthermore, the bulk expression data of PerSorted dim and lit cells revealed a significant downregulation (permutation *P* value < 0.05) of the genes whose protein products carry out glycolysis (see Data Set S1 in the supplemental material). In addition, the expression of citrate synthase (MSMEG_4035) is downregulated by 38-fold (*P* value = 0.006). Repression of this critical gene in the TCA cycle suggests that dim cells have reduced flux through the TCA cycle and exist in a low-O_2_ respiratory state. Finally, genes and processes associated with the action of multiple antibiotics were significantly downregulated within the dim cell population. For instance, MSMEG_3729 (*katG*), which encodes a catalase that converts INH into its active form was downregulated by ∼60-fold (*P* value < 0.0005), the ATP synthase F1 subunit (MSMEG_4936) targeted by bedaquiline was downregulated by 2.5-fold (*P* value < 0.001), and two cytochrome P450 genes targeted by azole drugs were also significantly downregulated (MSMEG_6030, 64-fold, *P* value = 0.003; MSMEG_1431, 15-fold, *P* value = 0.004) in the dim cell population. The downregulation of these antibiotic targets within dim cells together with their translationally dormant, low-O_2_, and slow-growing state both explains why they are phenotypically INH and RIF tolerant and also predicts that this subpopulation of cells is in fact multidrug tolerant.

10.1128/mSystems.01127-20.10 DATA SET S1 Table of differential expression (log_2_) of all genes and glycolysis-associated genes in PerSorted bulk dim cells. Download Data Set S1, XLSX file, 0.02 MB. Copyright © 2020 Srinivas et al. 2020 Srinivas et al. This content is distributed under the terms of the Creative Commons Attribution 4.0 International license.

### Activation of oxidative metabolism eliminates translationally dormant mycobacteria and achieves faster killing by INH and RIF in nutrient-starved conditions.

**FIG 5:**
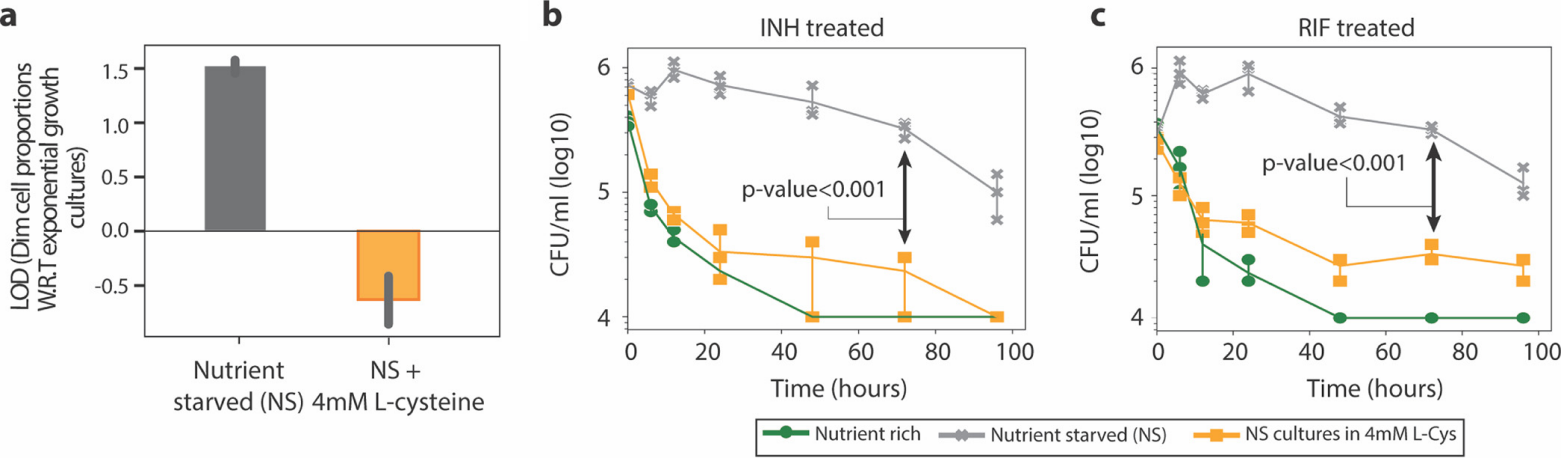
Dim cell proportion and time-kill curves upon l-cysteine addition to nutrient-starved M. smegmatis. (a) Proportion of dim cells in MSM-mEos2 cultures grown in nutrient-starved conditions with or without the addition of 4 mM l-cysteine. Dim cell proportions (log 10) under each condition were calculated with respect to MSM-mEos2 cultures grown in naive conditions. Error bars were calculated by measuring standard deviations in dim cell proportions between 10 PerSort replicates. (b and c) Time-kill curves of MSM cultures grown in nutrient-starved conditions with 4 mM l-cysteine and treated with 5× MIC INH (b) or 5× MIC RIF (c). Time-kill curve data for nutrient-rich and nutrient-starved conditions (no l-cysteine) are from Fig. 1. Data points are averaged from three independent experiments, and error bars represent the standard deviations between replicates. The limit of quantification for reporting growth was 10^3^ CFU/ml. Mean CFU/ml at 72 h between l-cysteine-treated and untreated cultures in nutrient-starved conditions were compared with Student's *t* test. LOD, log difference; W.R.T, with respect to.

Since respiration may play an important role in generating and/or maintaining translationally dormant mycobacteria, we explored the use of l-cysteine to activate oxidative metabolism and reduce the proportion of dim cells. It is well-known that exogenous amino acids fuel the TCA cycle and oxidative respiration in bacteria, especially molecules such as cysteine and proline which are rapidly metabolized (38). Cultures of MSM-mEos2 were grown in standard 7H9 medium with 4 mM l-cysteine (similar growth observed in 7H9 medium with or without 4 mM l-cysteine [data not shown]), induced with ATc, and PerSorted for dim and lit subpopulations. The l-cysteine-treated MSM-mEos2 cultures were found to be completely devoid of dim cells (Fig. 5a). Further, we supplemented nutrient-starved MSM cultures with 4 mM l-cysteine and then treated with either 5× MIC INH or 5× MIC RIF. Kill curves for the antibiotic- and l-cysteine-treated cultures were generated as previously described (Fig. 5b and c). Activation of respiration by adding l-cysteine to nutrient-starved cultures potentiated clearance by INH and RIF at rates identical to or better than rates for nutrient-rich cultures. This further corroborates that, similar to dim cells, the expanded translationally dormant mycobacteria in nutrient-starved conditions share a low respiratory physiological state. Ultimately, these results demonstrate that drug adjuvants that activate respiration could shorten drug treatment, particularly in environmental niches that foster drug tolerance in mycobacteria.

## DISCUSSION

The ability of microorganisms to survive sudden environmental changes stems from the formation of phenotypically heterogeneous subpopulations. Phenotypic heterogeneity confers fitness advantage to clonal microbial communities, such as infectious MTB, but impedes efforts of the immune system to clear the pathogen as well as chemotherapeutic efforts to rapidly treat TB. In this study, we developed a method to identify and characterize multidrug-tolerant subpopulations of mycobacterium cells. We demonstrated that our method enables a better understanding of phenotypic heterogeneity in mycobacteria in naive and stressed conditions and could lead toward novel strategies to shorten TB treatment.

We generated a fluorescent reporter system, PerSort, which sorts mycobacterial cells based on translational activity. We found a small proportion of translationally dormant (“dim”) cells from naive cultures, in the absence of stress such as antibiotic treatment. We confirmed that the translationally dormant subpopulation was tolerant to both INH and RIF, demonstrated a longer lag phase upon regrowth, and could regenerate the original population structure upon regrowth (i.e., a mixture of translationally active and dormant cells). These data indicate that the translationally dormant subpopulation identified by PerSort consists of cells with the properties of persisters. We report that these cells preexist in low numbers in an isogenic MSM culture growing without stress and in exponential phase of growth. The translationally dormant subpopulation (along with other phenotypically heterogeneous subpopulations) is generated stochastically, as a bet-hedging strategy, for the mycobacterial population to withstand unpredictable environmental stress.

Indeed, we discovered an increase in the proportion of the translationally dormant subpopulation under density-dependent stress, and even more so under nutrient starvation. This suggests an environmentally induced component that forms this subpopulation, in addition to stochastic formation. It also begs the question of what is influencing the translational state of this mycobacterial subpopulation. The PerSort method overcomes the challenge of characterizing persisters at single-cell resolution by sorting them and, importantly, not killing the susceptible cells with antibiotics. This technological advancement has overcome the confounding issue that antibiotic treatment itself induces persister cell formation (39, 40) and allows comparative analysis of persister cells and actively growing drug susceptible cells from the same culture. Because of these novel capabilities of PerSort, we were able to quantify transcript abundance of 45 genes associated with persister formation and drug tolerance in single cells from both the translationally dormant (i.e., dim) and translationally active (i.e., lit) subpopulations. Single-cell expression analysis revealed that translationally dormant persisters are a mix of *vapC30*, *mazF*, and *relA*/*spoT* overexpressing cells. These results reinforce the hypothesis that there are multiple pathways (both stochastically and deterministically activated) to become a persister cell and reveals the complex and combinatorial schemes used by mycobacteria to generate heterogeneous subpopulations.

Within the translationally dormant single cells, we found that high expression of toxin *mazF* was also associated with high *relA*/*spoT* expression. We suspect that *mazEF* could be regulated by the alarmone response, elicited by RelA/SpoT synthesis of (p)ppGpp, in a manner that is induced deterministically by stress conditions (41). In contrast, the single-cell expression data suggest that *vapC30* overexpression can also act to induce persister formation in a spontaneous and alarmone-independent manner. This supports a recent study demonstrating that a *relA*/*spoT* knockout mutant of MSM, with reduced alarmone response, still formed persisters at levels similar to those of the wild type (42). This collection of evidence points toward multiple mechanisms of generating the translationally dormant mycobacteria characterized here, some of which are deterministically activated (controlled by *relA*/*spoT* in response to stress) and some of which are stochastically activated (controlled by spontaneous *vapC30* activation) (43). While MSM has a single VapBC-type TA system, MTB has 70 copies of VapBC (32), indicating that the human pathogen has evolved to increase phenotypic diversity and bet-hedging for survival in the host environment. Much work is still needed, specifically using live-cell monitoring techniques to understand how and when these TA systems are activated to form persisters and the contribution of other mechanisms, either stochastic or deterministic, to phenotypic heterogeneity in MTB.

**FIG 6:**
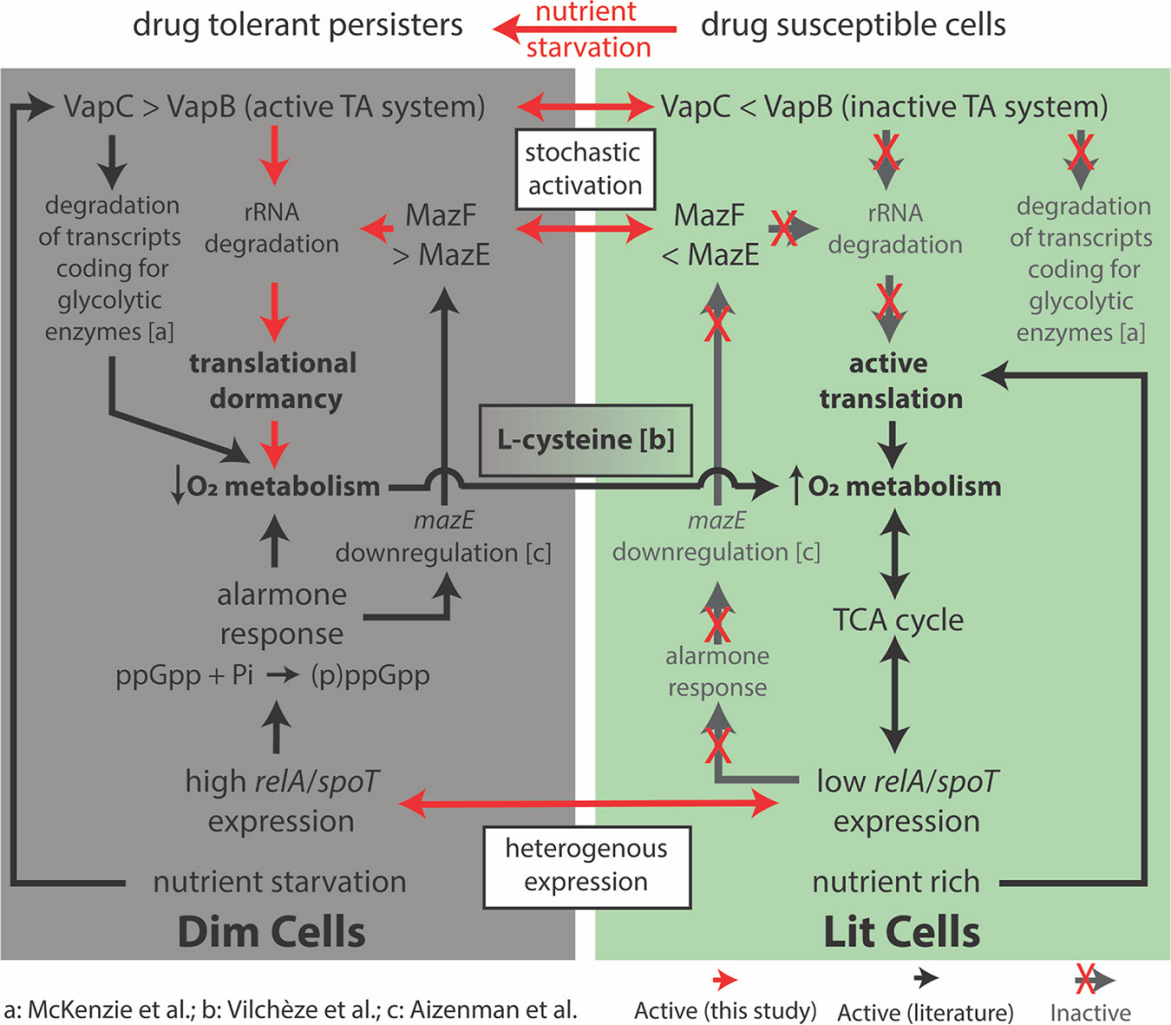
The persister subpopulation converges on a low-O_2_ metabolic state, which was exploited by addition of l-cysteine to convert the subpopulation to drug-susceptible cells. The diagram depicts the processes driving dim (multidrug-tolerant persisters) and lit (drug-susceptible) cell formation, as discovered through single-cell expression analysis. Evidence from the literature further connects these various mechanisms to the O_2_ respiratory state of either dim or lit cells. Red arrows indicate connections identified in this study, black arrows are previously determined connections from the literature (references indicated), and white boxes are proposed mechanisms for persister cell formation. The references from the literature indicated in the figure are McKenzie et al. (44), Vilchèze et al. (46), and Aizenman et al. (41).

The activation of TA systems (44) and the alarmone response (45) has been shown to decrease oxidative metabolism in bacterial persisters. Moreover, it is expected that translational dormancy would be associated with low respiration (25). Given this knowledge, we demonstrated lower ROS levels and reduced expression of glycolysis and TCA cycle genes in the translationally dormant subpopulation compared to translationally active cells, indicating a shift away from oxidative metabolism in the persister cells. In other words, regardless of their mechanism of formation (i.e., *vapC30*, *mazF*, or *relA*/*spoT* overexpression), the persister subpopulation shares a low-oxygen respiratory state, which presents a vulnerability that could be targeted to modulate the whole subpopulation (Fig. 6). Moreover, in addition to the translational dormancy, slow-growing, and low-O_2_ respiratory state, the downregulation of targets of multiple antibiotics predicts that the persister population is phenotypically multidrug tolerant. This is striking for two reasons: first, these persisters were not selected by treatment with any antibiotic, and second, targeting an antibiotic-specific tolerance mechanism is unlikely to achieve clearance of the persisters. In contrast, targeting a phenotype that is shared across the heterogeneous persister population, such as activation of oxidative metabolism, could potentiate the action of multiple drugs. Indeed, we confirmed that the addition of l-cysteine, which is known to activate oxidative metabolism (46), dramatically reduced the proportion of translationally dormant cells in nutrient-starved MSM cultures, conditions where the population is abundant, potentiating the action of both RIF and INH. This goes beyond previous studies by directly demonstrating that promoting oxidative metabolism reduces the proportion of multidrug-tolerant persister cells that form independent of drug pressure. Furthermore, the addition of l-cysteine was able to effect clearance by INH and RIF in nutrient-starved conditions. The addition of l-cysteine potentiates faster drug killing by converting (or limiting the formation of) multidrug-tolerant persister cells, cells with enhanced fitness advantage in the host-relevant stress (nutrient-starved) conditions to a population with increased oxidative metabolism and drug susceptibility. Our findings prove that until properties of heterogeneous subpopulations are disrupted, we will not be able to successfully clear mycobacterial cells in infected patients. This study highlights how novel methods to isolate and characterize heterogeneous subpopulations can enable targeted strategies to eliminate detrimental persister cells, and thereby shorten the course of treatment.

## MATERIALS AND METHODS

### Bacterial growth and MSM-mEos2 strain development.

Mycobacterium smegmatis (MSM) mc^2^155 strain was obtained from ATCC and grown in 7H9 broth medium (Difco) with 0.2% glycerol, 0.05% Tween 80, and 10% ADC enrichment (BD Biosciences). pSTKi-mEos2 plasmid was constructed from pSTKi plasmid and pRSETa mEos2 plasmid (22, 47). Synthetic oligonucleotide with translation initiation signal (mycoSD) was used to amplify mEos2 from pRSETa mEos2 plasmid, and amplified fragment was inserted into the pSTKi plasmid with restriction ligation at BamHI and EcoRI sites. The pSTKi-mEos2 transcript was electroporated into electrocompetent MSM cultures, and transformed colonies were selected on 7H10 plates with 30 μg/ml kanamycin (KAN). MSM-mEos2 cells were cultured in 7H9 medium supplemented with 30 μg/ml KAN.

### Development of PerSort.

The PerSort method was developed using BD FACS Influx. A 70-μm tip and sheath fluid from BD Biosciences was used in sorting and FACS analysis. Green fluorescent beads (3.5 μm) and 5-μm Accudrop beads from BD Biosciences were used to calibrate the instrument for laser alignment, compensation, and cell sorting. Propidium iodide (PI) stain (SigmaAldridge, 0.5%) was used to stain dead cells. Heat-killed cells (incubated at 70 to 80°C for 5 min) were used as dead cell control for PI stain. MSM cells with pCHERRY3 plasmid (MSM-mCherry) (2) was used to optimize single-cell mycobacterium sorting. MSM-mEos2 (KAN-resistant) cultures were grown to an OD_600_ of 0.6 and induced with 500 ng/ml anhydrotetracycline (ATc). Induced cultures were incubated at 37°C for 12 h. MSM-mCherry (hygromycin [HYG]-resistant) cultures were mixed with induced MSM-mEos2 cultures in equal proportions, and single mEos2 and mCherry cells were sorted and plated on 7H10 plates with KAN and HYG to determine the efficiency of single-cell sorting (see Fig. S2 in the supplemental material). Gates for sorting dim and lit cells ATc-induced MSM-mEos2 strains (induced for 12 h) were determined using uninduced MSM-mEos2 strains stained with PI. FACS data were analyzed in FlowJo software (ver. 10).

### Antimicrobial tolerance assay.

MICs of the MSM-mEOS2 strains were determined with a disk diffusion assay (3). Drug-treated bacteria were washed with or diluted (10^−3^ and 10^−5^) in 7H9 medium and plated (100 μl) or spotted (5 μl) on 7H10 medium with 30 μg/ml KAN at time points 0 to 92 h after incubation. Percentage survival was calculated as a relative percentage of CFU observed in the sample with respect to the 0-h time point.

### qRT-PCR of PerSorted samples.

Dim and lit cells were sorted into 500 μl TRIzol, and RNA was extracted with DirectZol RNA purification kit (Zymo Research) by the manufacturer’s instructions. Quantitative reverse transcription-PCR (qRT-PCR) was performed with Luna Universal Probe one-step RT-qPCR (real-time quantitative PCR) kit (New England Biolabs). Log_2_ normalized RNA abundances were calculated using ValidPrime signal as reference (see Table S1 in the supplemental material) (4). Primers AGAGTTTGATCCTGGCTC (forward) and GCCATGCGACCAGCAG (reverse), GGATGCCTTGGCACTG (forward) and AGACGCCTATATATTCAGC (reverse), and ACATCACCGAAACTCCCG (forward) and GTCGACGAGCAGGTCCA (reverse) were used to determine expression of 16S rRNA, 23S rRNA, and phosphoglucoisomerase (PGI) (MSMEG_5541), respectively.

### Bulk transcriptome sequencing (RNAseq) of PerSorted samples.

Dim and lit cells (*n* = 300,000) were PerSorted into 1 ml TRIzol having ∼1,000 bone marrow-derived macrophage cells. RNA was extracted by the phenol chloroform method described previously (41), rRNA was depleted with Illumina rRNA depletion kit, and mycobacterium RNA was enriched with Path-seq methodology described previously (41). A total of 100,240 probes were designed to cover M. smegmatis mc^2^155 (Agilent probe library ELID number S3206492). Differential expression in dim cells was calculated with DESeq (48) using mean expression of lit cells as reference.

### Single-cell persister gene expression and analysis.

The Fluidigm Biomark system with 48 × 48 plates was used for this study. Single dim and lit cells were PerSorted into 96-well plates with VILO reaction mix (5×), SUPERase (Invitrogen), and 10% NP-40 in a prenoted random order to avoid sampling bias. Sorted plates were spun down and freeze-thawed three times on dry ice to rupture cells. ValidPrime assay (4) for nontranscribed genomic DNA was used to determine the rupture efficiency and calculate the signal from a single nucleic acid strand (used as reference to calculate transcript abundance). Reverse transcription (RT) was performed on freeze-thawed cells with VILO cDNA preparation mixture, T4-Gene32 protein, and random hexamer primers. RNA spike-in (ECC2_SpikeIn RNA, 10 pM) was included in the RT master mix. cDNA of the genes of interest (Table S1) was preamplified with TaqMan PreAmp master mix (Invitrogen) and an equimolar mixture of forward and reverse strand primers designed for the genes of interest (Table S1). Primers were removed with exonuclease I (ExoI) (Invitrogen). Primer sets used for preamplification were primed into the 48 × 48 Biomark assay plates. Quantitative PCR assay with Biomark prescribed protocol was run on diluted, preamplified ExoI-treated cDNA with Sso Fast EvaGreen Supermix (Bio-Rad Laboratories). Quality control for determining, sorting, cell lysis, and cDNA preparation was performed by comparing the threshold cycle (*C_T_*) values of genomic DNA control and spike-in control in all the cells (Fig. S6a and b). Expression levels of genes were measured as Δ*C_T_* in individual cells with reference to genomic DNA control (expected to result from one copy of genomic DNA), less than or equal expression (of genomic DNA control) was considered zero expression, and assays with *C_T_* of >40 were flagged as missing values. Δ*C_T_* values for each cells were corrected by adding or subtracting the deviation from the median Δ*C_T_* of the spike-in control for a particular cell. Hierarchical clustering was performed with Python Seaborn package and the R package, PVclust.

### Feature selection of single-cell clusters.

The ratio of toxin expression to their respective antitoxin expression was calculated and included along with the other persister gene expression values. Key features that differentiate dim and lit subpopulations were selected with tree-based feature selection tool (5). Weights of the features that signify its ability to differentiate between dim and lit sub populations were estimated as an average over 100 iterations of randomly selected subsets of the data set (70% of gene expression values). Constraints of gene regulation were also used in selection of features, as most persister-specific responses are a part of alarmone response, a broad, genome-wide change in transcript levels (6). The three features with the highest weights and evidence of additional criteria were selected for identification of clusters within dim and lit cells.

### *In situ* ROS level measurements with CellRox assay.

MSM-mEos2 cells were induced with ATc (500 ng/ml) and incubated for 12 h under standard growth conditions. *N*-Acetyl cysteine (NAC) (10 μM), an oxidative agent that reduces the level of *in situ* reactive oxygen species (ROS), was added to the induced MSM-mEos2 cultures and incubated at 37°C for 90 min to prepare the negative control. Along with the negative control, another set of induced cultures were treated with a ROS-inducing compound, 100 μM MnTBAP (catalog no. 55266-18-7; Sigma-Aldrich) and incubated for 15 min to prepare the positive control. CellRox orange (catalog no. C10443; Thermo Fisher), was added to the induced samples and control and incubated for 30 min at room temperature. FACS analysis was performed to measure the CellROX orange intensity. ROS-positive and -negative gates were determined with control samples, and ROS levels in dim and lit cell population were determined with reference to the controls.

### Dim cell proportion and antimicrobial tolerance following l-cysteine addition.

MSM-mEos2 cells were grown in PBS and 0.05% Tween 80, with and without 4 mM l-cysteine, to an OD_600_ of 0.6. Cultures were induced with ATc (500 ng/ml) and incubated for 12 h. Dim cell proportions in both conditions were measured with PerSort assay and normalized to the dim cell proportions observed in optimal growth conditions (growth in 7H9 medium without l-cysteine). Tolerance of cultures grown in various conditions was measured with time-kill curve assays, under 5× MIC RIF (17.5 μg/ml) and 5× MIC INH (20 μg/ml) treatment. The percent survival was measured at various time points by counting the number of CFU on 7H10 plates in reference to the zero time point of the respective samples.

### Data availability.

Data from bulk RNAseq of PerSorted dim and lit samples are available in Gene Expression Omnibus database under accession number GSE160767.
