# Supplementary figures and images for: PerSort Facilitates Characterization and Elimination of Persister Subpopulation in Mycobacteria
Source: mSystems. 2020 Dec 1;5(6):e01127-20. doi: 10.1128/mSystems.01127-20 (PMC7716392; doi:10.1128/mSystems.01127-20)

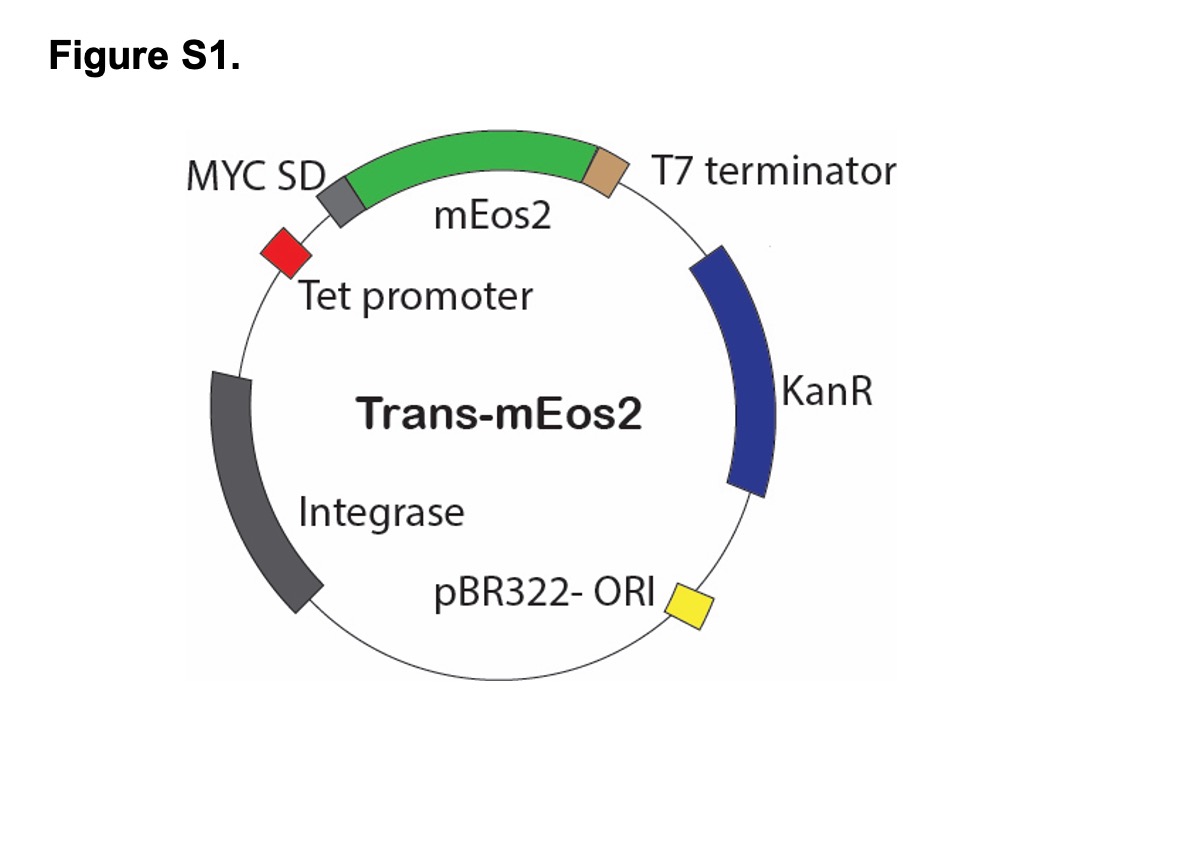

Supplement: FIG S1 [file mSystems.01127-20-sf001.jpg]

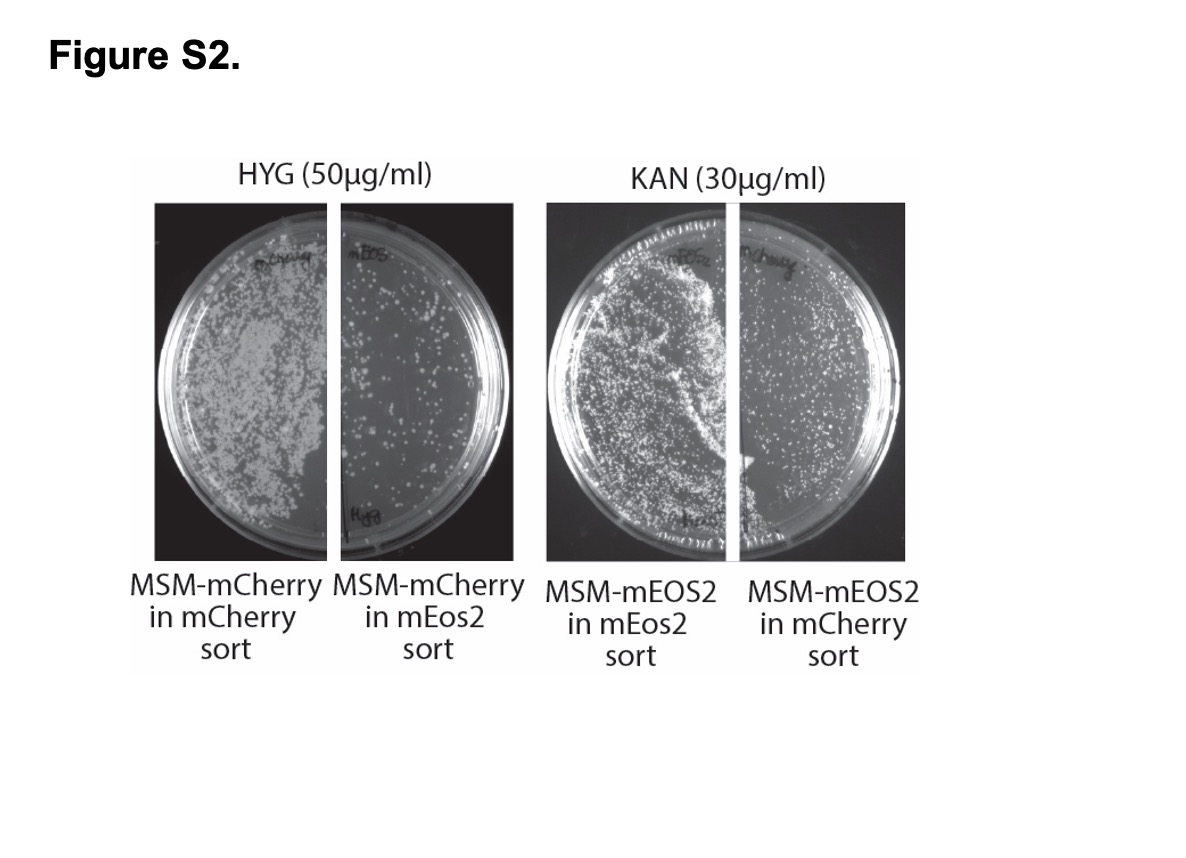

Supplement: FIG S2 [file mSystems.01127-20-sf002.jpg]

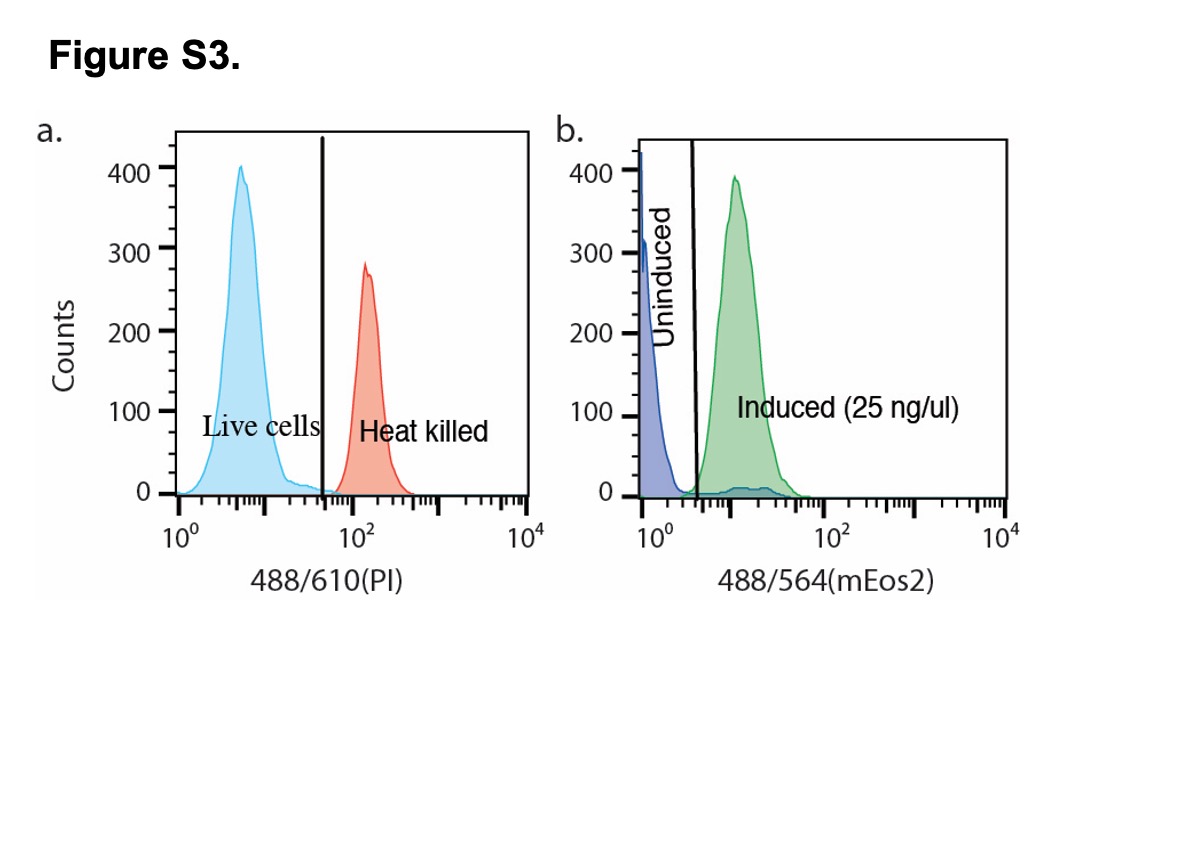

Supplement: FIG S3 [file mSystems.01127-20-sf003.jpg]

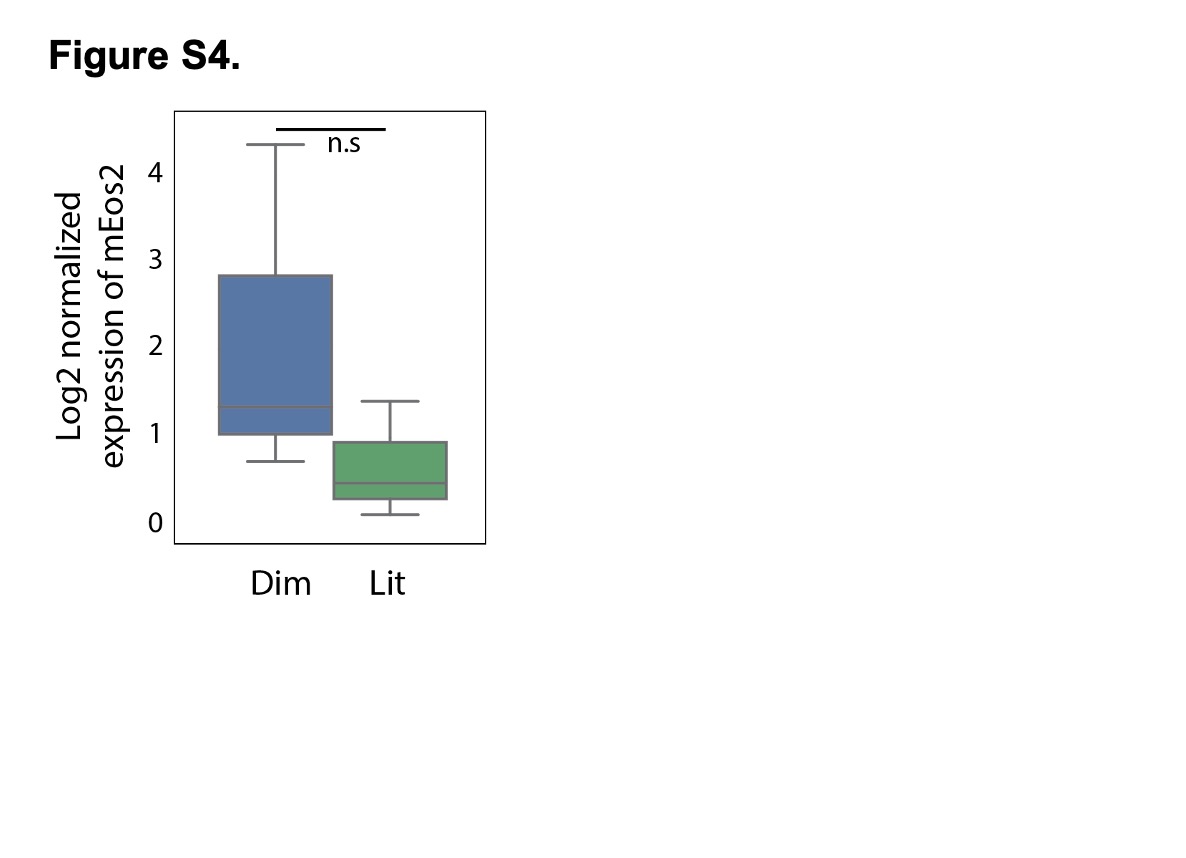

Supplement: FIG S4 [file mSystems.01127-20-sf004.jpg]

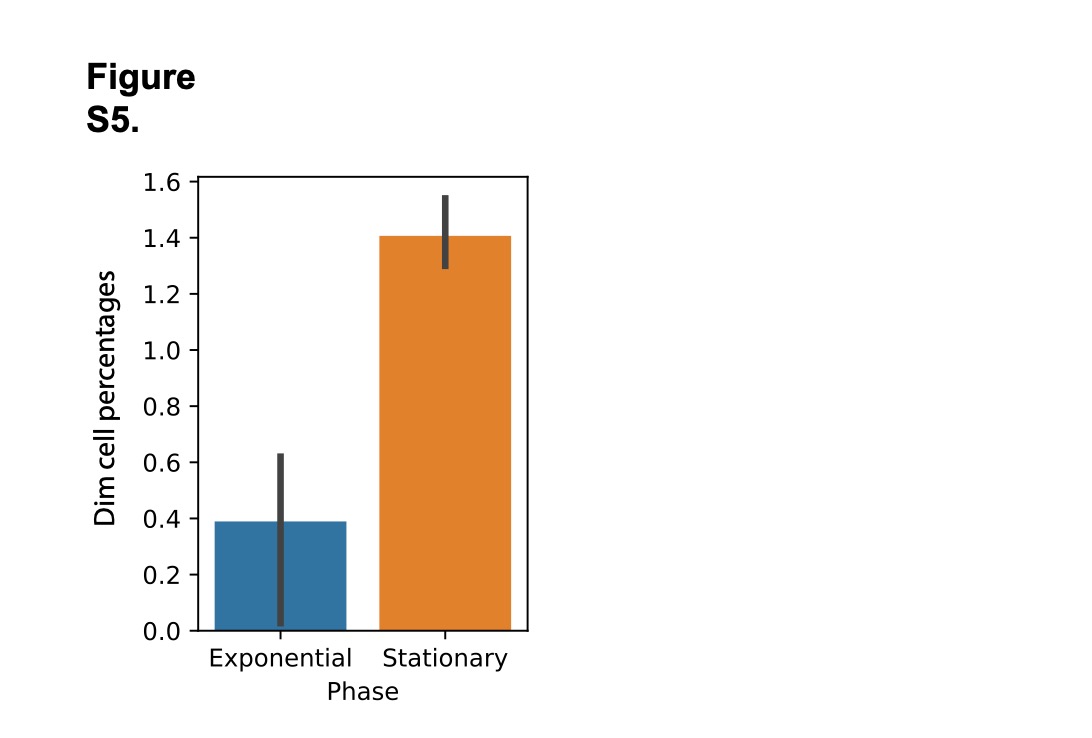

Supplement: FIG S5 [file mSystems.01127-20-sf005.jpg]

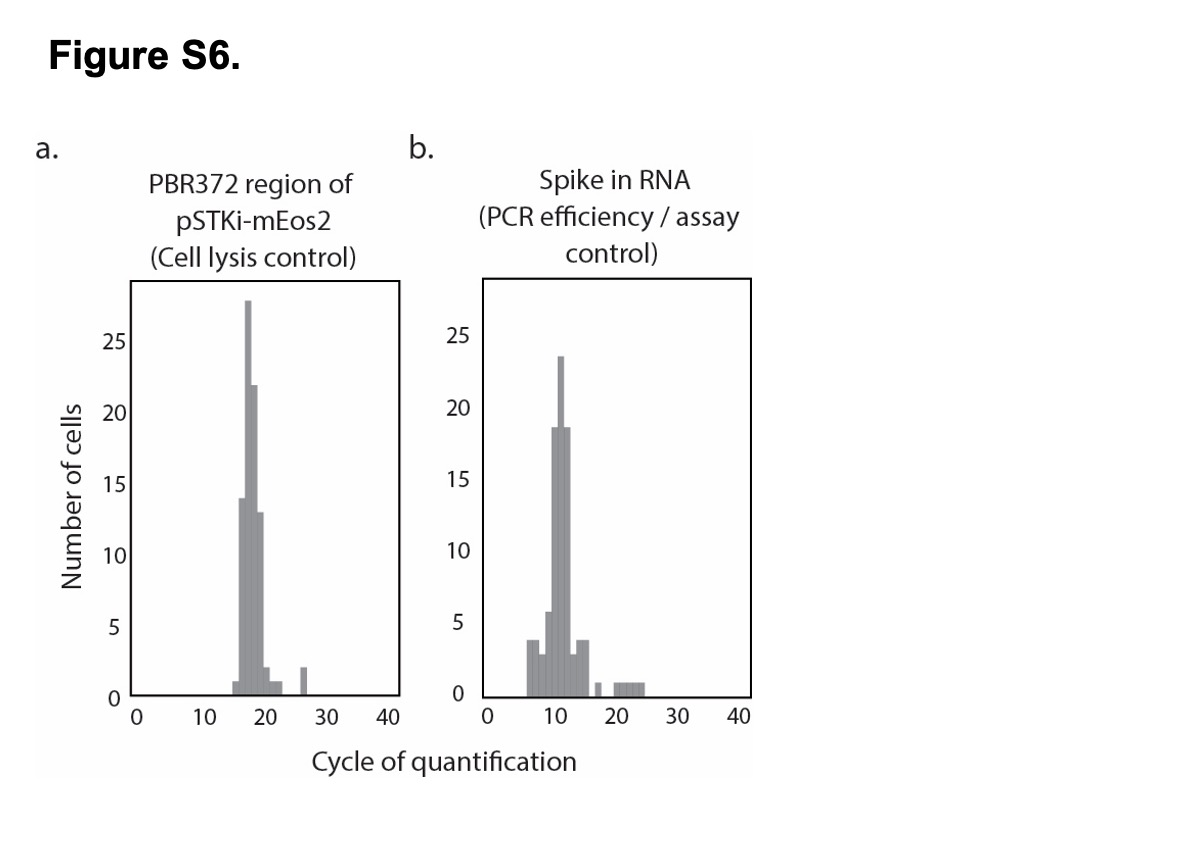

Supplement: FIG S6 [file mSystems.01127-20-sf006.jpg]

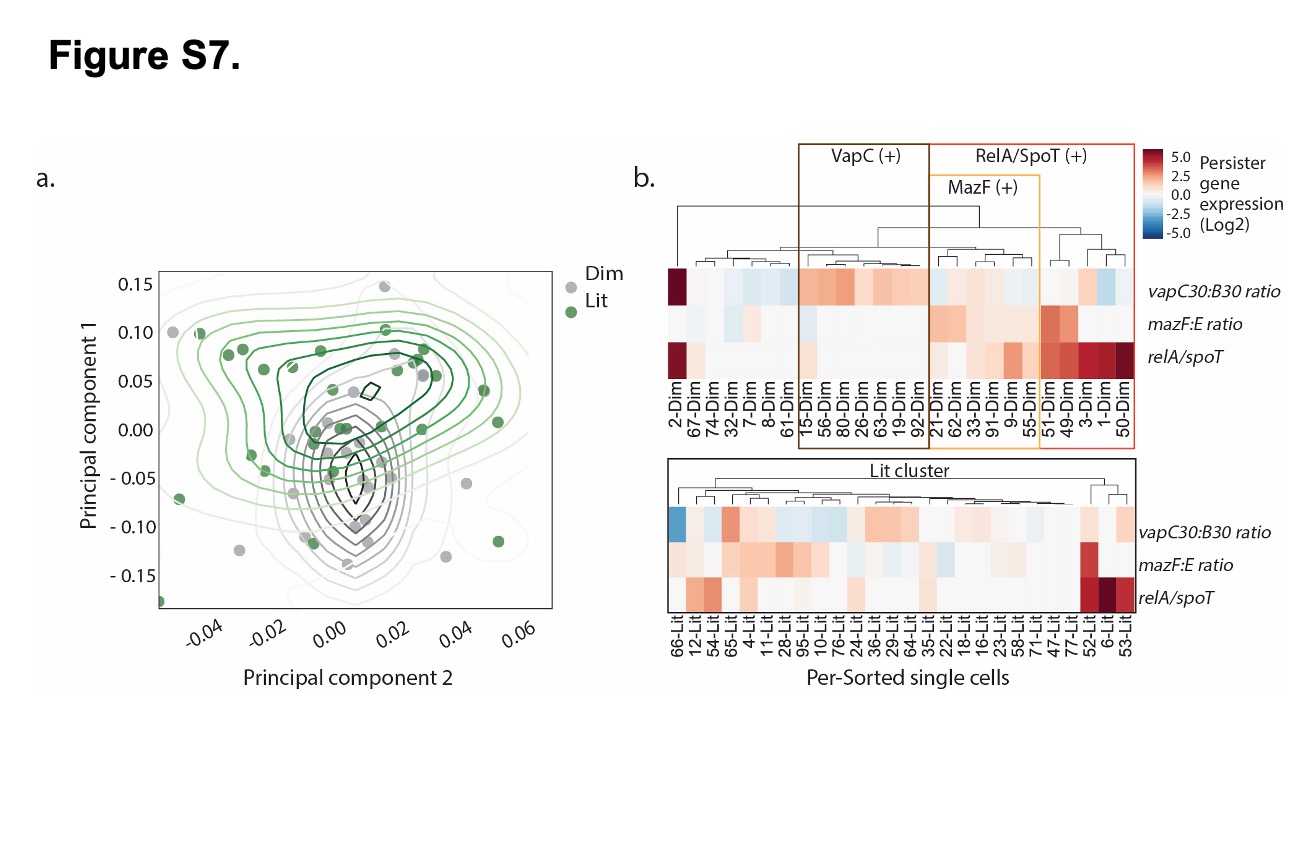

Supplement: FIG S7 [file mSystems.01127-20-sf007.jpg]

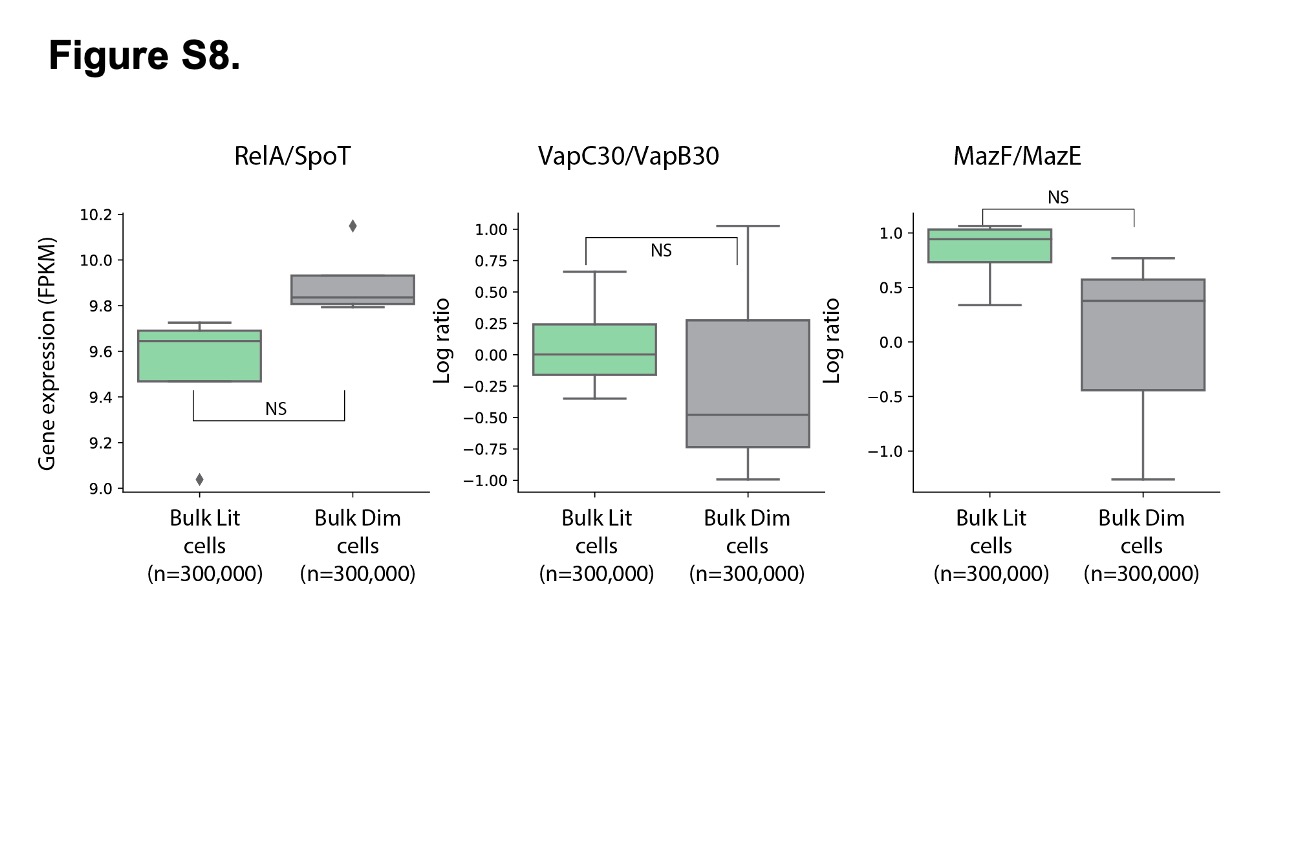

Supplement: FIG S8 [file mSystems.01127-20-sf008.jpg]
