# Supplementary material for: PerSort Facilitates Characterization and Elimination of Persister Subpopulation in Mycobacteria
Source: mSystems. 2020 Dec 1;5(6):e01127-20. doi: 10.1128/mSystems.01127-20 (PMC7716392; doi:10.1128/mSystems.01127-20)
Supplement: TABLE S1 [file mSystems.01127-20-st001.docx]

**Table S1.**

| **MSM gene ID** | **Gene name** | **Gene function** | **Reference** | **Weights** | **Forward Primer** | **Reverse Primer** |
| --- | --- | --- | --- | --- | --- | --- |
| *MSEMG_2740* | *lexA* | Alarmone response | (1,2) | 0.0216 | GACACCGGCGAGTTCACGGA | TCGAGGATGGTGCGCTGACG |
| *MSMEG_0424* | *hsp* | Persister phenotype associated gene | (1,2) | 0.0577 | AATCCGACGGCCGCACCTAC | CCGGCGACCCGTACCTTCAG |
| *MSMEG_0709* | *dnaK* | Regulator | (1,2) | 0.0216 | GCGACCTCCGGTGACAACCA | GATGCCCGAGCTGCCCTTGA |
| *MSMEG_0713* | *hspR* | Sigma factor | (1,2,5) | 0.0431 | ACCTGCTGCGAGAGGTGCAG | AGCGCGTCGACCTGATTGGT |
| *MSMEG_0913* | *umaA* | Persister phenotype associated gene | (1,2) | 0.0077 | GGAAGTCCGCCTGCAGGGTT | GGGTAGCGCTCGGCCTTGAA |
| *MSMEG_0916* | *madR* | Transcriptional regulator | (1,2) | NA | CCGTCGGCGGTGTCCAGCTC | ATGCCGCACAGCGCGTACCA |
| *MSMEG_1277* |  | TA operon | (1,2) | 0.0163 | GGCGGATGACCTGTCGCTGA | GGCGACCCTGCGCTTGTG |
| *MSMEG_1278* |  | TA operon | (1,2) | NA | CCATGCGCTGGTCGACGGTA | TCCTCCGCGCGATCGACATC |
| *MSMEG_1283* | *vapB30* (Antitoxin) | TA operon | (1,2,6,7) | 0.0216 | GAGGCGGTGGTGATGGCACT | GAGGCGGTGGTGATGGCACT |
| *MSMEG_1284* | *vapC30* (Toxin) | TA operon | (1,2,6,7) | 0.0648 | GCGGTGGCTGACGATCCTGT | GAGTTCGCGACCACCTGGCT |
| *MSMEG_1605* | *phoY1* | Persister phenotype associated gene | (1,2) | NA | CCGATCTGACGCTGGCCGAA | GTGCATGGACCCGACGACCA |
| *MSMEG_1633* | *dnaE2* | DNA replication | (1,2,4) | 0.0162 | AGTGGGCCCGCATGGAGAAC | CCGAGGCCCAGCATGTCGAA |
| *MSMEG_1803* | *rsbW* | Persister phenotype associated gene | (1,2) | 0.0576 | GTGGATCCCGGTCCCGATGC | CAGGCCCGGACCCATCTGTG |
| *MSMEG_1914* | *rpoE1* | Sigma factor | (1,2,5) |  | GTCCAACGCCGAGCACTCCT | GCTTGCAACGCGGCCTTGAT |
| *MSMEG_1915* | *rshA* | Persister phenotype associated gene | (1,2) | 0 | GCCTGCGGCATTACGGCATC | TCGTGGTGCGGCTGATCTGG |
| *MSMEG_2389* | *mdp1* | Regulator | (1,2) | 0.0369 | CACAGAAGCTCCCGGCCGAT | CACAGAAGCTCCCGGCCGAT |
| *MSMEG_2391* | *ppk1* | Alarmone response | (1,2) | 0.0043 | GCTGTTGGAGCGCGCGAAAT | AGCGCACCGACAGACCCATC |
| *MSMEG_2723* | *recA* | Recombination enzyme | (1,2) | NA | CAGGCGCTGCGCAAGATGAC | CTCGGGCGAGCCGAACATCA |
| *MSMEG_2752* | *sigB* | Sigma factor | (1,2,5) | 0.0167 | TCGACATGCCGGTCGGAACC | GGCGGACATGGCCTCGGAAT |
| *MSMEG_2758* | *mysA* | Sigma factor | (1,2,5) | 0.0108 | AGGGCGAGAAGCTGCCAGTG | GCAGGTTCGCCTCCAGCAGA |
| *MSMEG_2817* | ABC efflux pump 1 | Alarmone response | (1,2) | 0.0216 | TGGGAGCCGCTGGCTTCTAC | CCGACGACGGTACCGAGGAA |
| *MSMEG_2965* | *relA/spoT* | Effector of alarmone response | (1–3) | 0.0717 | GTGCTCGCCGACGAGAAGGT | GTGCTTCGGGTCGCCCATCT |
| *MSMEG_3151* | *inhA* | Drug target | (1,2) | 0 | TCGACGGTGTGGTGCACTCG | GCGCGTCGAAGAACGGGTTG |
| *MSMEG_3180* | antitoxin | TA operon | (1,2) | NA | TCGCGGTGCTCATGGACGAC | CGGGCACGTGCACTGCATTC |
| *MSMEG_3181* | Toxin | TA operon | (1,2) | 0.0214 | CCGGACGCGGTCTACGTGTT | AAGCTCACCCGCACGATCCC |
| *MSMEG_3944* | *devR c1* | Metabolic control | (1,2) | 0.0474 | CGACCCGAAGTCGCGGTTCT | GTGGCCTCGTCGGACGTGAA |
| *MSMEG_3945* | ABC transporter3 | Alarmone response | (1,2) | NA | ATCCACGGCGAGTCGAAGGC | CGTCGAGAGGCTGCGTCGAA |
| *MSMEG_4175* | *arsR* (Antitoxin) 1 | TA operon | (1,2) | 0.0129 | TGCCCTGGTCGACGGTGAAC | GACCTCGCGCAGCACCTTGA |
| *MSMEG_4176* | *arsR* (Toxin) 1 | TA operon | (1,2) | NA | GGCACGGTGCTTCGCTTCAC | CGGTCGAAGAAGGCGTGGGT |
| *MSMEG_4265* | *lamA/mmpS3* | Persister phenotype associated gene | (1,2) | 0.0234 | GCCGACGTGGCGCTCTATGA | GCCGACGTGGCGCTCTATGA |
| *MSMEG_4427* | efflux pump 1 | Efflux pump 1 | (1,2) | 0.0207 | GCGGTTTGGCTTCCGCAGTC | GCGGCGCTGACCTTCAACAC |
| *MSMEG_4447* | *aze* (Antitoxin) | TA operon | (1,2) | NA | ACCGAGTACGCCGACATCGC | GGCGGCGACCAACTCAGACT |
| *MSMEG_4447* | *mazE* (Antitoxin) | TA operon | (1,2) | 0.0249 | AATCGAGCCAACGCCAGCCA | CGACACGGTGCGTCAAGCTG |
| *MSMEG_4448* | *mazF* (Toxin) | TA operon | (1,2) | 0.0173 | CGGTTCCGCCACATCACCCT | GGTGAGCGCGTAGACGGTGT |
| *MSMEG_4466* | *uspA* | Persister phenotype associated gene | (1,2) | 0 | GATCGCCCGCAAGAGCGAGA | GACGCCTGCGTACCCTCCAG |
| *MSMEG_5141* | *narK2* | Persister phenotype associated gene | (1,2) | NA | GGTCGGATCGTTGGGACGCA | CAGGATCGACGCGACCGTCA |
| *MSMEG_5244* | *devR c2* | Metabolic control | (1,2) | NA | TCACCCAGCAGGAGCGTGTG | CGCGCCGCGATCTGTTTGTT |
| *MSMEG_5248* | *desA2* | Desaturase | (1,2) | 0.0162 | GCGGGCCTCGACGTGATCGG | CTCGGCGACGTTGGCGACCT |
| *MSMEG_5659* | ABC transporter1 | Alarmone response | (1,2) | 0.0062 | TGGCGTCTCGGCCTGATGTG | TACGTGCGGGCGGATTCGTT |
| *MSMEG_5660* | ABC transporter2 | Alarmone response | (1,2) | 0.0664 | GTCGACCATCCGCCGGTTCA | CAGCAGGATCGCGGTGACCA |
| *MSMEG_5773* | *desA1* | Desaturase | (1,2) | 0.0609 | GGCCTCGACATCGCGCCGAA | GCGGAGCACCGGCATCACGA |
| *MSMEG_5776* | *phoY2* | Persister phenotype associated gene | (1,2) | 0 | CACGCGGGATCCGGAGAAGG | CCACTCGCGGTCCATCAGCA |
| *MSMEG_6225* | proton antiporter efflux pump | Alarmone response | (1,2) | 0.0984 | GGGTGCCGTGGTGTCGATGA | CCAGGCCCGTGACCATCAGG |
| *MSMEG_6384* | *katG* | Metabolic control | (1,2) | NA | CCGGTGAGCGTGACCTGGAG | TGCGGATCCGGATTGCCGTT |
| *MSMEG_6760* | *arsR* (Toxin) 2 | TA operon | (1,2) | NA | CCCGGACGGCGAGAAGTACG | AGCGAACCCGTCGAGGAACG |
| *MSMEG_6762* | *arsR* (Antitoxin) 2 | TA operon | (1,2) | 0.0173 | CACGAGGCGCGACATCATGC | AGCAGGCCGGCTTTCTCCAG |
| *NA* | *vapC30:B30* | NA | NA | 0.0371 | NA | NA |
| *NA* | *mazF:E* | NA | NA | 0.0378 | *NA* | *NA* |
| *NA* | Assay control (Spike in) | NA | NA | NA | TCCAGATTACTTCCATTTCCGC | GCTGGATGCCGACGCCCGTAT |
| *NA* | Genomic DNA control or Valid prime control (PBR372 region of pSTKi mEos2) | NA | NA | NA | TGGCTGCTGCCAGTGGCGAT | GCCCGACCGCTGCGCCTTAT |

**References**

1. Torrey HL, Keren I, Via LE, Lee JS, Lewis K. High Persister Mutants in Mycobacterium tuberculosis. PLoS ONE [Internet]. 2016 May 13 [cited 2017 Oct 2];11(5). Available from: https://www.ncbi.nlm.nih.gov/pmc/articles/PMC4866775/

2. Keren I, Shah D, Spoering A, Kaldalu N, Lewis K. Specialized Persister Cells and the Mechanism of Multidrug Tolerance in Escherichia coli. J Bacteriol. 2004 Dec 15;186(24):8172–80.

3. Dutta NK, Klinkenberg LG, Vazquez M-J, Segura-Carro D, Colmenarejo G, Ramon F, et al. Inhibiting the stringent response blocks Mycobacterium tuberculosis entry into quiescence and reduces persistence. Sci Adv. 2019 Mar 1;5(3):eaav2104.

4. Henry TC, Brynildsen MP. Development of Persister-FACSeq: a method to massively parallelize quantification of persister physiology and its heterogeneity. Sci Rep. 2016 May 4;6:srep25100.

5. Pisu D, Provvedi R, Espinosa DM, Payan JB, Boldrin F, Palù G, et al. The Alternative Sigma Factors SigE and SigB Are Involved in Tolerance and Persistence to Antitubercular Drugs. Antimicrob Agents Chemother. 2017 Dec 1;61(12):e01596-17.

6. McKenzie JL, Robson J, Berney M, Smith TC, Ruthe A, Gardner PP, et al. A VapBC toxin-antitoxin module is a posttranscriptional regulator of metabolic flux in mycobacteria. J Bacteriol. 2012 May;194(9):2189–204.

7. Ahidjo BA, Kuhnert D, McKenzie JL, Machowski EE, Gordhan BG, Arcus V, et al. VapC Toxins from Mycobacterium tuberculosis Are Ribonucleases that Differentially Inhibit Growth and Are Neutralized by Cognate VapB Antitoxins. PLOS ONE. 2011 Jun 29;6(6):e21738.
